# Imaging the Bacterial Cell Wall Using *N*-Acetyl Muramic Acid-Derived Positron Emission Tomography Radiotracers

**DOI:** 10.1021/acssensors.3c01477

**Published:** 2023-11-22

**Authors:** Sang Hee Lee, Jung Min Kim, Marina López-Álvarez, Chao Wang, Alexandre M. Sorlin, Kondapa Naidu Bobba, Priamo A. Pichardo-González, Joseph Blecha, Youngho Seo, Robert R. Flavell, Joanne Engel, Michael A. Ohliger, David M. Wilson

**Affiliations:** † Department of Radiology and Biomedical Imaging, University of California, San Francisco, San Francisco, California 94158, United States; ‡ UCSF Helen Diller Family Comprehensive Cancer Center, University of California, San Francisco, San Francisco, California 94158, United States; § Department of Pharmaceutical Chemistry, University of California, San Francisco, San Francisco, California 94158, United States; ∥ Department of Medicine, University of California, San Francisco, San Francisco, California 94158, United States; ⊥ Department of Microbiology and Immunology, University of California, San Francisco, San Francisco, California 94158, United States; # Department of Radiology, Zuckerberg San Francisco General Hospital, San Francisco, California 94110, United States

**Keywords:** infection imaging, peptidoglycan, *N*-acetyl muramic acid, positron emission tomography, fluorine-18

## Abstract

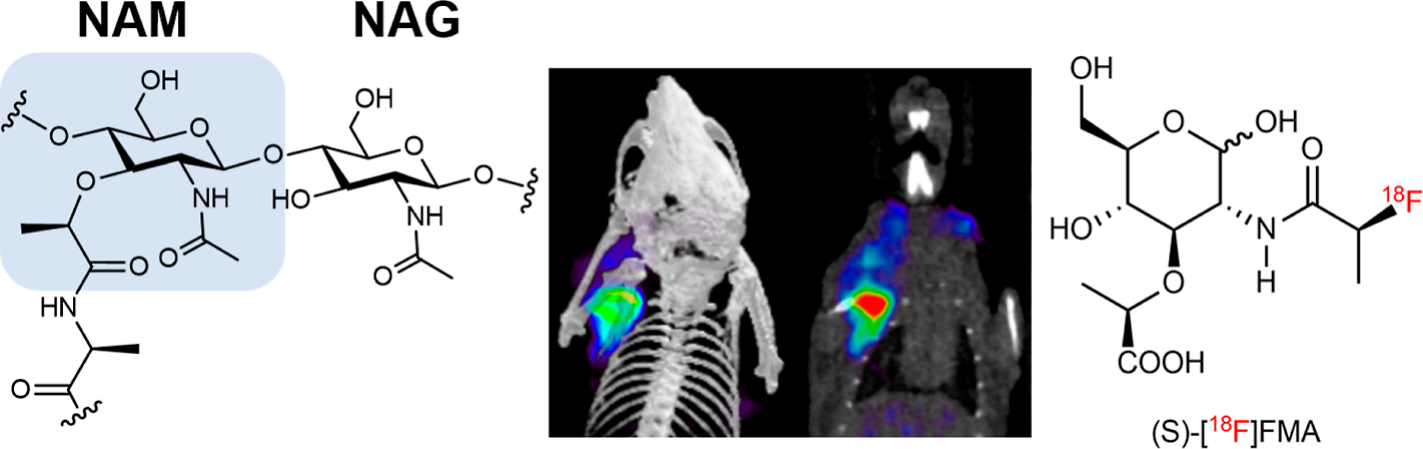

Imaging infections in patients is challenging using conventional methods, motivating the development of positron emission tomography (PET) radiotracers targeting bacteria-specific metabolic pathways. Numerous techniques have focused on the bacterial cell wall, although peptidoglycan-targeted PET tracers have been generally limited to the short-lived carbon-11 radioisotope (*t*_1/2_ = 20.4 min). In this article, we developed and tested new tools for infection imaging using an amino sugar component of peptidoglycan, namely, derivatives of *N*-acetyl muramic acid (NAM) labeled with the longer-lived fluorine-18 (*t*_1/2_ = 109.6 min) radioisotope. Muramic acid was reacted directly with 4-nitrophenyl 2-[^18^F]fluoropropionate ([^18^F]NFP) to afford the enantiomeric NAM derivatives (*S*)-[^18^F]FMA and (*R*)-[^18^F]FMA. Both diastereomers were easily isolated and showed robust accumulation by human pathogens in vitro and in vivo, including *Staphylococcus aureus*. These results form the basis for future clinical studies using fluorine-18-labeled NAM-derived PET radiotracers.

In the past decade, several imaging methods have been developed that explicitly target bacteria-specific metabolic pathways, using clinically translatable technologies, such as positron emission tomography (PET) or magnetic resonance imaging (MRI). Some of the most promising PET radiotracers include radiolabeled sugars and sugar alcohols that are not efficiently metabolized by humans, such as 2-deoxy-2-[^18^F]fluoro-d-sorbitol ([^18^F]FDS).^1,2^ These methods show strong clinical potential, but many are limited in detecting Gram-positive organisms, including *Staphylococcus aureus*. For example, the most clinically advanced radiotracer, [^18^F]FDS, accumulates in Gram-negative *Enterobacteriaceae* only. This selectivity could represent an advantage for determining organism type (and tailoring appropriate antimicrobial therapy) but represents a limitation for detecting infections caused by other bacteria. We and other groups have therefore sought *S. aureus*-sensitive radiotracers for the study of orthopedic and cardiovascular infections, motivating the development of the peptidoglycan-targeted probes d-[methyl-^11^C]methionine^3−5^ and d-[3-^11^C]alanine^6^ (Figure 1). A major limitation of these d-amino acid-derived tracers is the short half-life of carbon-11 (20 min), which would limit their application in the acute care setting (i.e., emergency room, hospital) based on the need for an on-site cyclotron and difficult logistics related to radiosynthesis, quality control, patient transport, and technologist use. Most importantly, many acutely ill patients are treated at facilities lacking a cyclotron.

**Figure 1:**
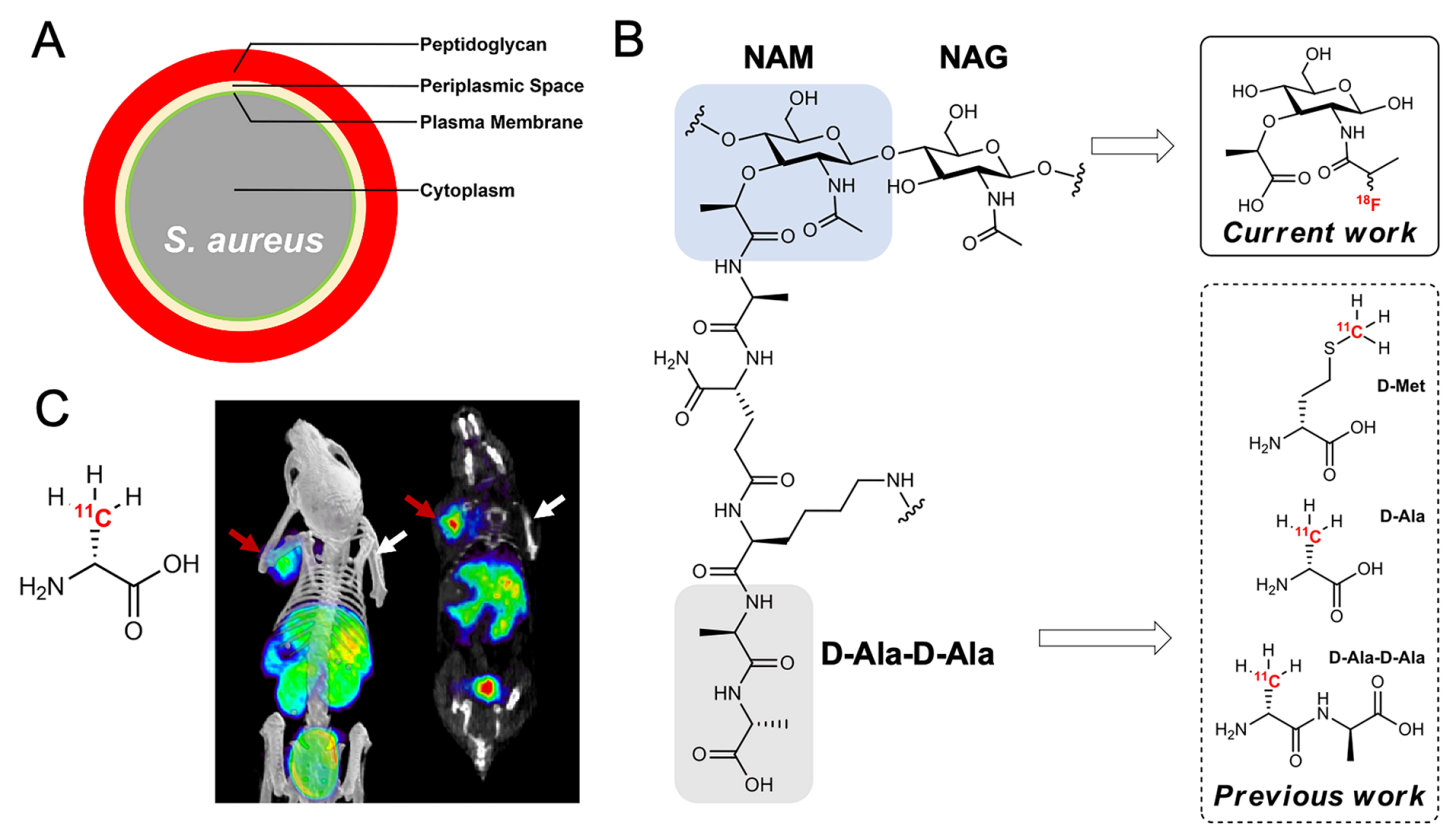
Radiolabeled molecules targeting the bacterial cell wall for PET imaging. (A) Depiction of the bacterial cell wall. (B) Chemical structure of the S. aureus peptidoglycan monomer (PGM) consisting of *N*-acetylglucosamine (NAG) and *N*-acetylmuramic acid (NAM) with a pentapeptide (l-Ala, d-*iso*-Gln, l-Lys, and d-Ala–d-Ala). (C) Representative PET/CT image from our previous work using d-[^11^C]Ala in a murine model of acute bacterial infection (red arrow) with heat-killed bacteria (white arrow). Reproduced with permission from Parker et al.^6^ Copyright 2020 American Chemical Society.

Therefore, a critical goal is development of an ^18^F-labeled bacteria-specific tracer with (1) a straightforward radiosynthesis and (2) sensitivity to Gram-positive organisms including *S. aureus*. Based on the literature and our own experience, modifications of the d-amino acid scaffold present steric challenges for peptidoglycan incorporation. For example, using fluorescent d-amino acid-derived probes, Fura et al. showed a clear relationship between side-chain size and labeling of the bacterial surface suggesting that resemblance to the canonical muropeptide residues d-alanine and d-glutamate is critical.^7^ We have therefore looked beyond the peptide component of peptidoglycan and toward its carbohydrate backbone, specifically *N*-acetyl muramic acid (NAM).^8^ Together with *N*-acetyl glucosamine (NAG), NAM is a core structural element of peptidoglycan with numerous modifications reported for cell wall labeling, including azide/alkyne derivatives of the *N*-acetyl moiety for subsequent bioorthogonal detection.^9^ As described in several elegant publications by the Grimes group, in engineered *Escherichia coli* these NAM tags were tolerated by the recycling enzymes MurNac/GlcNAc anomeric kinase (AmgK) and NAM α-1 phosphate uridylyl transferase (MurU) allowing formation of the modified UDP NAM for visualization of peptidoglycan via copper-catalyzed azide–alkyne cycloaddition (CuAAC).^10,11^ Although potential mechanisms of modified NAM incorporation are not reported or fully understood for other bacteria, we hypothesized that a variety of pathogens might also be able to incorporate exogenous NAM derivatives. Therefore, analogous to the previous Grimes work, ^18^F-NAM derivatives synthesized via *N*-acylation were potentially a straightforward approach for microbial detection using PET.

A major advantage of *N*-acylation for labeling carbohydrate derivatives is the direct use of nonprotected amino sugars and amino sugar alcohols. An ^18^F-modified derivative of *N*-acetyl muramic acid could potentially be synthesized via an SN_2_ displacement of a protected electrophile using [^18^F]fluoride, analogous to the radiosynthesis of the widely used 2-deoxy-2-[^18^F]fluoro-d-glucose ([^18^F]FDG).^12^ We hypothesized that for the precious muramic acid (>$20 per mg), acylation via an ^18^F-labeled activated ester would furnish the desired NAM derivative, a method that could be applied to many commercially available amino sugars/sugar alcohols. For the selective acylation of amines in PET, the most commonly used reagent is *N*-succinimidyl-4-[^18^F]fluorobenzoate ([^18^F]SFB).^13^ However, *N*-benzoylation is not a typical modification found in nature. In contrast, *N*-acetylation is observed in numerous biologic processes, including epigenetic remodeling of histones^14^ and post-translational changes of key bacterial proteins.^15^ To radiolabel NAM while minimizing structural perturbations, we sought an *N*-acylation method that closely mimicked acetate, with the understanding that (1) a carbon-11 acetylation method while compelling would not yield a bacteria-sensitive tracer with the desired half-life and (2) reports using ^18^F-fluoroacetate suggest significant defluorination in vivo. Fujiwara et al. described the analogous *N*-[^18^F]fluoroacetyl-d-glucosamine with imaging in a murine tumor model showing moderate bone uptake.^16^ We therefore decided to acylate muramic acid with 4-nitrophenyl 2-[^18^F]fluoropropionate ([^18^F]NFP),^17,18^ to validate the NAM-derived diastereomers (*S*)-[^18^F]FMA and (*R*)-[^18^F]FMA for imaging bacterial infection. Successful use of *N*-acylation for NAM radiolabeling would also motivate new methods for ^18^F-incorporation into NAc mimics that can be applied to various metabolites of biochemical/medicinal interest.

## Experimental Section

### General

All chemical reagents were purchased from Sigma-Aldrich and Chem Scene and used without further purification. Reactions were monitored using precoated silica gel plates (Merck, silica gel 60 F_254_). Flash column chromatography was performed on silica gel (Merck, 230–400 mesh). ^1^H and ^13^C NMR spectra were obtained on a Bruker Avance III HD 400 MHz instrument at the UCSF Nuclear Magnetic Resonance Laboratory. High-resolution mass spectroscopy (HRMS) services were provided by the University of California, Berkeley Spectrometry Facility. All bacterial strains were purchased from American Type Culture Collection (ATCC) except *S. aureusXen29*, *E. coliXen14*, and *Pseudomonas aeruginosaXen41*, which were purchased from PerkinElmer and a methicillin-resistant *S. aureus* clinical isolate which was provided by the University of Nebraska Medical Center. [^18^F]fluoride ion was generated in the UCSF radiopharmaceutical facility by the nuclear reaction of ^18^O(p,n)^18^F in a target of enriched [^18^O]H_2_O using a PET trace 18 MeV cyclotron (GE Healthcare, Buckinghamshire U.K.). Radio TLC analysis was performed on a radio TLC scanner (Bioscan AR200, Bioscan Inc.). PET/CT imaging of mice was performed by a Siemens Inveon micro-PET/CT (Siemens, Erlangen, Germany). The radioactivity for in vitro and ex vivo analyses was measured on a Hidex Automatic Gamma Counter (Turku, Finland).

### Radiochemistry

[^18^F]fluoride ion (3 mL in enriched [^18^O]H_2_O) was passed through the female side of a Chromafix-HCO_3_ cartridge (ABX advanced biochemical compounds GmbH, Radeberg, Germany) preconditioned with 2 mL of EtOH and 8 mL of water. The retained [^18^F]fluoride ion was eluted into a reaction vial (4 mL) from the male side of the cartridge using a solution of Cs_2_CO_3_ (2.2 mg, 6.8 μmol) in 90% MeOH/water (1 mL). The solvent was removed under an N_2_ stream with reduced pressure at 90 °C. Azeotropic distillation of the mixture with ^18^F ion was performed using acetonitrile (1.0 mL × 2) under the same conditions. 4-Nitrophenyl-2-bromopropanoate (8 mg, 29.2 μmol) dissolved in 80% *t*BuOH/MeCN (0.5 mL) was added to the reaction vial and then stirred at 110 °C for 10 min. After the reaction, the crude mixture was cooled in an ice bath and diluted with 20 mL of water. The mixed solution was passed through a tC18 Sep-Pak cartridge, and then eluted with MeCN (1.5 mL). The crude mixture eluted from the cartridge was diluted with water (1.5 mL) and injected into an HPLC system (Phenomenex, Luna 10 μm C18 column, 250 × 10 mm; 45% MeCN/water containing 0.1% trifluoroacetic acid; and λ = 254 nm, flow rate = 4.0 mL min^–1^) to collect the [^18^F]NFP (*T*_R_ = 19 min). [^18^F]NFP in HPLC eluent (typically 4–5 mL) was diluted with 18 mL of water and then loaded into the tC18 Sep-Pak cartridge. Then, the female side of the Sep-Pak dry sodium sulfate Plus Long Cartridge (without preconditioning) was connected to the male side of a tC18 Sep-Pak Cartridge containing [^18^F]NFP. An additional 2 mL of Et_2_O was passed through the female side of the cartridges connected in series. The collected solution of [^18^F]NFP in a 4 mL vial was dried under an N_2_ stream under reduced pressure at room temperature. After complete drying, muramic acid (0.25 mg, 1.0 μmol) dissolved in 100 μL of DMSO containing 0.1% TEA (v/v) was added to the reaction vial and heated at 60 °C for 10 min. The mixture was diluted by 5% EtOH/water containing 0.1% HCl (0.9 mL), then injected into the HPLC system (Phenomenex, Luna 10 μm C18 column, 250 × 10 mm; 5% EtOH/water containing 0.1% HCl; λ = 210 nm, and flow rate = 4.0 mL min^–1^) to collect the (*S*)-[^18^F]FMA (*T*_R_ = 16.3 min) and (*R*)-[^18^F]FMA (*T*_R_ = 19.9 min), respectively. Both (*R*)- and (*S*)-[^18^F]FMA in the HPLC eluent (typically 4–5 mL) were diluted with 18 mL of acetonitrile, and then loaded onto a Plus NH_2_ Sep-Pak cartridge. Finally (*R*)- and (*S*)-[^18^F]FMA were eluted using saline (1.5 mL), ready for in vitro or in vivo studies. The radiochemical purities of both (*R*)- and (*S*)-[^18^F]FMA were confirmed by analytical HPLC (Phenomenex, Luna 10 μm C18 column, 250 × 4.6 mm; 5% EtOH/water containing 0.1% HCl; λ = 210 nm, and flow rate = 1.0 mL min^–1^).

### In Vitro Stability Assays

Saline, mouse serum, or human serum (0.5 mL) was incubated with (*S*)- and (*R*)-[^18^F]FMA (3.7 MBq), respectively, at 37 °C for 0, 15, 30, 60, and 90 min (*n* = 3 for each time point). Each sample was diluted with acetonitrile (0.5 mL) and then centrifuged at 3500 rpm for 5 min. The collected supernatants were analyzed using radio thin-layer chromatography (radio-TLC) by developing at 65% MeCN/water.

### In Vitro Uptake Assays

Each bacterial strain was aerobically grown overnight in a shaking incubator at 37 °C in media summarized in Supporting Information, Table S1. Overnight cultures were diluted to an optical density at 600 nm (OD_600_) of 0.05 and grown to exponential phase (∼0.4). In vitro uptake assays were conducted by incubating bacteria cultures with 30 μL from the stock solution of (*S*)- and (*R*)-[^18^F]FMA (29.6 MBq/mL) at 37 °C for 90 min. As controls, heat-killed bacteria (pretreated at 90 °C for 30 min) were incubated in the same conditions with radiotracers. The bacterial cultures were incubated with unlabeled muramic acid (0.1 mM) and radiotracers under the same conditions for the blocking experiments. Aliquots of bacterial cultures (300 μL) were centrifuged at 13,200 rpm for 6 min and washed with PBS (300 μL). The radioactivity of the pellets and supernatants was measured using a gamma counter (Hidex, Turku, Finland). The in vitro data were normalized to cfus to account for differential growth rates between organisms. The nonspecific binding of both tracers in the filter membrane was measured by incubating (*S*)- and (*R*)-[^18^F]FMA in the media without bacteria at 37 °C for 90 min.

### Animals for PET Imaging Studies

All animal studies were approved by the Institutional Animal Care and Use Committee at UCSF and performed in accordance with the UCSF guidelines. CBA/J mice (female, 9–11 weeks old, 20–24 g) were used for all experiments. All of the animals were anesthetized with 5% isoflurane during infection and μPET/CT imaging. The murine myositis model used was generated according to our previous protocol^6^ by inoculating with 50 μL of *S. aureus*, *E. coli*, or *Staphylococcus epidermidis* (∼10^6^ cfu) in the left shoulder muscle and 10X heat-killed (pretreated at 90 °C for 30 min) bacteria in the right shoulder muscle. The infections were allowed to develop for 10 h prior to PET imaging. Upon completion of μPET/CT imaging, mice were sacrificed immediately for biodistribution analysis. The radioactivity accumulated in harvested tissues was measured by using a Hidex Automatic Gamma Counter (Hidex, Turku, Finland).

### μPET/CT Imaging Studies

The μPET/CT imaging studies were conducted by using a Siemens Inveon micro-PET-CT scanner (Siemens, Erlangen, Germany). For all studies, whole-body dynamic PET images of healthy or infected mice (*n* = 4 for each) were obtained with 53 frames (2s × 15, 5s × 6, 10s × 6, 30 s × 4, 60s × 6, and 300s × 16 frames, respectively) for 90 min immediately after injection of (*S*)- and (*R*)-[^18^F]FMA (7.4 ± 1.8 MBq, 100 μL) via a tail vein using a catheter, followed by a micro-CT scan for 10 min. All data were reconstructed into three-dimensional images to generate dynamic PET images and coregistered with CT images using open-source Amide software.

### Image Analysis

Amide software was used for analyzing image data.^19^ The volumes of interest (VOI’s) were drawn manually for each organ (brain, liver, left ventricular chamber, lung, kidneys, and bladder) to obtain a PET-derived biodistribution profile in healthy mice. Identical volumes and shapes (spherical, 5–8 mm^3^) of VOI’s were drawn around the peak uptake of tracers for the right and left shoulders of the bacteria-infected mice. Radioactivity in the VOI’s at each time point was expressed as the standardized uptake value (SUV), which is normalized to the injected radioactivity and body weight of mice and used to generate time–activity curves (TACs). The image-based blood TACs were generated based on the blood pool derived from LV chamber analysis. The kinetic parameters of radiotracers in each organ of healthy mice were calculated from TAC by fitting a biexponential curve using GraphPad Prism v9.0 software (GraphPad Software Inc., San Diego, California, USA) as following: distribution half-life (*T*_1/2α_), elimination half-life (*T*_1/2β_), peak concentration (*C*_max_), time at *C*_max_ (*T*_max_), and area under the curve (AUC).

### Statistical Analysis

All data were expressed as the mean ± standard deviations. Statistical analyses were conducted by an unpaired two-tailed Student’s *t*-test using GraphPad Prism v9.0. *P* < 0.05 was considered statistically significant.

## Results and Discussion

### Radiosynthesis and Characterization of (*S*)- and (*R*)-[^18^F]FMA

We initially considered incorporating fluorine-18 into the NAM via late-stage [^18^F]fluorination using protected-precursor, analogous to *N*-[^18^F]fluoroacetyl-d-glucosamine which was radiosynthesized using 1,3,4,6-tetra-*O*-acetyl-2-deoxy-2-bromoacetamido-d-glucopyranose.^20^ However, as previously stated, we decided to follow an alternative approach in this study using an amine-reactive ^18^F-prosthetic agent for time- and cost-effective radiosynthesis of ^18^F labeled *N*-acyl muramic acid. 4-Nitrophenyl-2-[^18^F]fluoropropionate ([^18^F]NFP) and *N*-succinimidyl-4-fluorobenzoate ([^18^F]SFB) are the most widely used amine-reactive prosthetic agents for labeling peptides under mild reaction conditions with a high labeling efficiency. Based on the previous reports from the Grimes group, we hypothesized that [^18^F]SFB was not appropriate for ^18^F-labeled NAM due to the steric size and absence of hydrogen at the α position of the amide carbonyl. For these reasons, we chose [^18^F]NFP for NAM labeling.

[^18^F]NFP was synthesized with a comparable radiochemical yield and molar activity to that previously reported by Haskali et al.^17^ [^18^F]NFP was obtained from 4-nitrophenyl-2-bromopropanoate in a 12.8 ± 3.4% radiochemical yield (*n* = 12, nondecay corrected) with high radiochemical purity (>99%) and molar activity (78.6 ± 32.2 GBq/μmol) after HPLC purification (Supporting Information, Figure S1A). The radiochemical identity of [^18^F]NFP was confirmed by coinjection with a ^19^F standard using analytical HPLC (Supporting Information, Figure S1B).

[^18^F]NFP was conjugated to α-muramic acid, which was dissolved in DMSO just before the coupling reaction to minimize mutarotation. Finally, α-(*S*)-[^18^F]FMA (*T*_R_ = 16.3 min) and α-(*R*)-[^18^F]FMA (*T*_R_ = 19.9 min) were collected, respectively, from the HPLC system with a 35.0 ± 3.6% radiochemical yield (nondecay corrected, calculated from the isolated activity of [^18^F]NFP), high radiochemical purity (>99%), and molar activity (57.4 ± 23.5 GBq/μmol) after formulation in saline using Plus NH_2_ Sep-Pak. The total synthesis time was 90 min. The stability of (*S*)- and (*R*)-[^18^F]FMA was verified in saline, mouse serum, and human serum at 37 °C at 0, 15, 30, 60, and 90 min using radio-TLC. Both (*S*)- and (*R*)-[^18^F]FMA were intact in saline, mouse serum, and human serum for 90 min (Supporting Information, Figure S2). Additionally, we attempted stereospecific radiosynthesis of [^18^F]FMA from an enantiopure *O*-tosylate precursor [(*S*)-NOTsP]. However, racemized [^18^F]FMA was observed by HPLC after the coupling reaction with α-MA probably due to loss of enantiopurity at higher reaction temperatures (Supporting Information, Figure S3).

**Figure 2:**
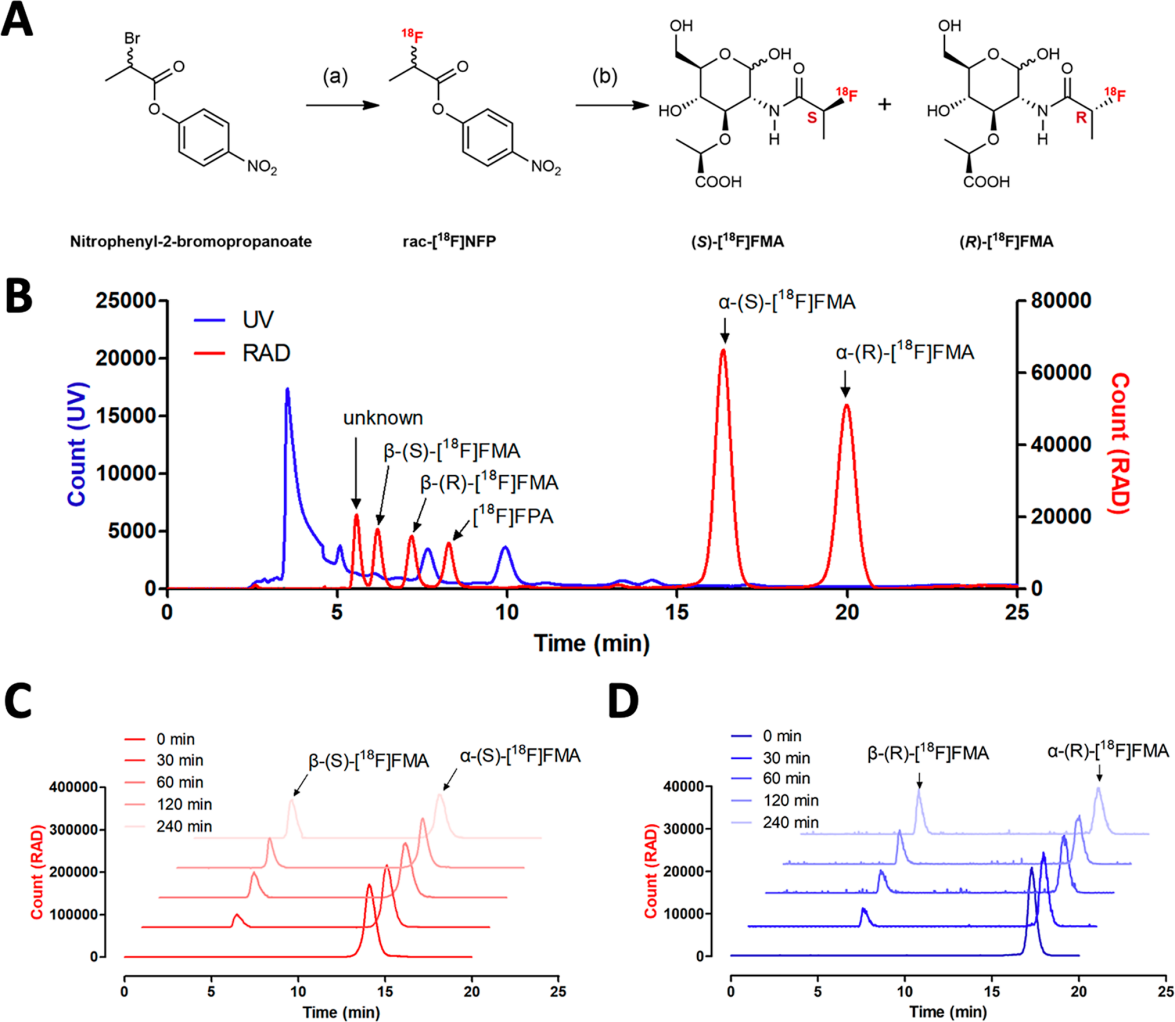
Radiosynthesis and characterization of (*S*)- and (*R*)-[^18^F]FMA. (A) Scheme for the radiosynthesis of (*S*)- and (*R*)-[^18^F]FMA using [^18^F]NFP. Reagents and conditions: (a) 4-nitrophenyl-2-bromopropanoate (8 mg, 29.2 μmol), ^18^F^–^, Cs_2_CO_3_ (2.2 mg, 6.8 μmol), 80% *t*BuOH/MeCN (0.5 mL), 110 °C, 10 min and (b) α-muramic acid (0.25 mg, 1.0 μmol), 0.1% TEA/DMSO (100 μL), 60 °C, 10 min. (B) Semipreparative HPLC profile (Phenomenex; Luna 10 μm C18 250 × 10 mm; 5% EtOH/water containing 0.1% HCl; 4 mL min^–1^) of the crude mixture of (*S*)- and (*R*)-[^18^F]FMA. Time-dependent α–β interconversion of (*S*)-[^18^F]FMA (C) and (*R*)-[^18^F]FMA (D) after HPLC purification (0, 30, 60, 120, and 240 min) at ambient conditions, monitored by analytical HPLC (Phenomenex; Luna 10 μm C18 250 × 4.6 mm; 5% EtOH/water containing 0.1% HCl; 1 mL min^–1^).

Because racemic [^18^F]NFP was used for the coupling reaction, four diastereomers were observed on HPLC chromatograms (*T*_R_ = 6.1, 7.1, 16.3, and 19.9 min) with hydrolyzed [^18^F]NFP (<5%, 2-[^18^F]fluoropropanoic acid, *T*_R_ = 8.2 min; confirmed by coinjection, see Supporting Information, Figure S4) and an unknown peak (<5%, *T*_R_ = 5.5 min), as shown in Figure 2B. Among the products, two major diastereomers [α-(*S*)- and α-(*R*)-[^18^F]FMA] were isolated, respectively, after the reaction. Even though we successfully isolated α-(*S*)- and α-(*R*)-[^18^F]FMA using HPLC, both diastereomers could naturally undergo mutarotation in aqueous solution. As expected, isolated α-(*S*)- and α-(*R*)-[^18^F]FMA showed α–β interconversion under ambient conditions, monitored by analytical HPLC for 0, 30, 60, 120, and 240 min. The α–β interconversions of α-(*S*)- and α-(*R*)-[^18^F]FMA were equilibrated to approximately 1.6(α)/1(β) ratio after 4 h (Figure 2C,D), confirmed by analytical HPLC with the corresponding ^19^F standards before and after equilibrium (Supporting Information, Figure S5A–D).

The equilibration of α–β sugars is a well-known process in aqueous solution at ambient conditions, which may be accelerated at high temperatures (>80 °C).^21^ To assess mutarotation of the α–β anomers, isolated α-(*S*)- and α-(*R*)-[^18^F]FMA were heated at 90 °C for 10 min to speed up the α–β interconversion and then analyzed by analytical-HPLC. After heating, the β anomers were generated from the corresponding α-(*S*)- and α-(*R*)-[^18^F]FMA. β-(*S*)- and β-(*R*)-[^18^F]FMA were isolated from the mixture using a semiprep HPLC system and confirmed by analytical-HPLC. Again, isolated β-(*S*)- and β-(*R*)-[^18^F]FMA were heated at 90 °C for 10 min and finally, we confirmed that α-(*S*)- and α-(*R*)-[^18^F]FMA were generated from the corresponding β-(*S*)- and β-(*R*)-[^18^F]FMA (Supporting Information, Figure S6A,B). In addition, to confirm the stereochemistry of the (*S*)- and (*R*)-[^18^F]FMA products, we synthesized ^19^F standards for both *R*,*S* diastereomers from the corresponding enantiopure 2-fluoropropanoic acids via 2 steps, as described in the Supporting Information (Scheme S1). Due to the short fluorine-18 half-life (109.8 min), all in vitro and in vivo studies were conducted immediately after HPLC purification of the corresponding α-anomers instead of preparing α,β equilibrated (*S*)- and (*R*)-[^18^F]FMA.

### Both (*S*)- and (*R*)-[^18^F]FMA Showed Robust Accumulation by Several Human Pathogens In Vitro Including Methicillin-Resistant *S. aureus*

**Figure 3:**
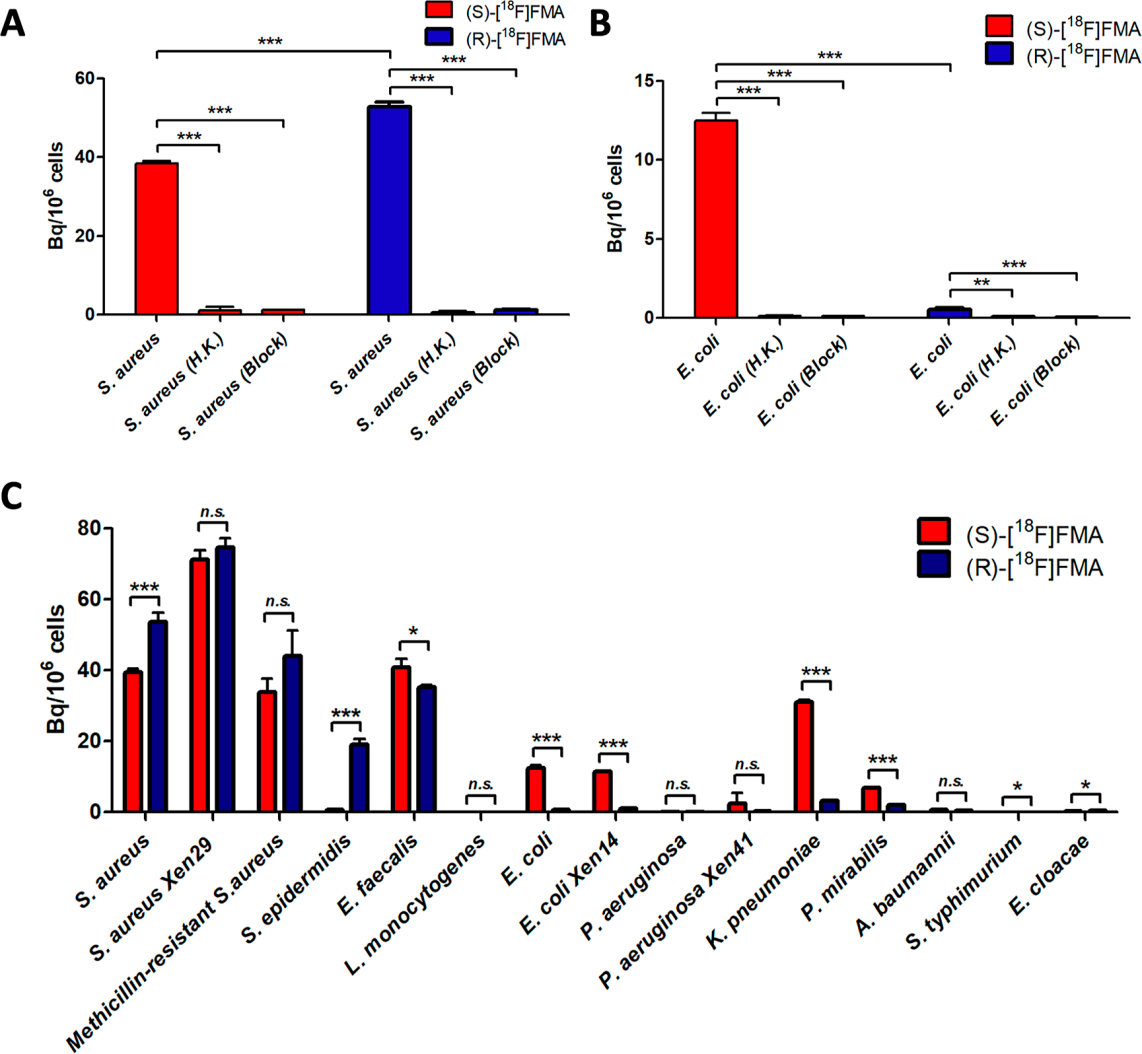
In vitro analyses of (*S*)-[^18^F]FMA and (*R*)-[^18^F]FMA in bacteria (*n* = 4 for each). In vitro cellular uptake of (*S*)-[^18^F]FMA and (*R*)-[^18^F]FMA in *S. aureus* (A) and *E. coli* (B). H.K.: heat-killed; block: blocked with *N*-acetyl muramic acid (0.1 mM). (C) Sensitivity analysis of (*S*)-[^18^F]FMA and (*R*)-[^18^F]FMA in different bacterial pathogens. *P < 0.05, **P < 0.01, ***P < 0.001, n.s.: not significant.

It is well known that stereochemistry has a marked impact on biological behavior, including cellular uptake, metabolic stability, and pharmacokinetic/dynamic profiles. We therefore investigated both (*S*)- and (*R*)-[^18^F]FMA in vitro and in vivo immediately after radiosynthesis and formulation to minimize α–β interconversion. We initially performed an in vitro uptake assay of (*S*)- and (*R*)-[^18^F]FMA in *S. aureus* and *E. coli*, key Gram-positive and negative bacterial pathogens, to evaluate the sensitivity and specificity of (*S*)- and (*R*)-[^18^F]FMA. A slightly higher uptake of (*R*)-[^18^F]FMA was observed in *S. aureus* versus that of (*S*)-[^18^F]FMA (1.4-fold, *P* < 0.0001). On the other hand, (*S*)-[^18^F]FMA showed a significantly higher uptake (23.1-fold, *P* < 0.0001) in *E. coli* than that of (*R*)-[^18^F]FMA. The specificity of both (*S*)- and (*R*)-[^18^F]FMA was demonstrated by incubating them with heat-killed or unlabeled “blocking” *N*-acetyl muramic acid (0.1 mM) in *S. aureus* and *E. coli*, respectively (Figure 3A,B). Based on the α–β interconversion of [^18^F]FMA, we conducted an in vitro assay in *S. aureus* after equilibration (4 h following formulation) to investigate the uptake efficiency of α versus β anomers. After α–β equilibration, slightly reduced uptake of (*S*)- (0.8-fold, *P* < 0.0080) and (*R*)-[^18^F]FMA (0.8-fold, *P* < 0.0012) in *S. aureus* was observed (Supporting Information Figure S7A,B). These results suggest that the incorporation of the α anomer is slightly better than that of the β anomer.

Next, the following Gram-positive and Gram-negative pathogens were screened to explore the sensitivity of (*S*)- and (*R*)-[^18^F]FMA: *S. aureus*, *S. aureusXen29*, methicillin-resistant *S. aureus* (MRSA), *S. epidermidis*, *Enterococcus faecalis*, *Listeria monocytogenes*, *E. coli*, *E. coliXen14*, *P. aeruginosa*, *P. aeruginosaXen41*, *Klebsiella pneumoniae*, *Proteus mirabilis*, *Acinetobacter baumannii*, *Salmonella typhimurium*, and *Enterobacter cloacae*. (*R*)-[^18^F]FMA showed a high uptake in Gram-positive bacteria pathogens except *L. monocytogenes* but a significantly lower uptake in Gram-negative pathogens. On the other hand, (*S*)-[^18^F]FMA showed higher sensitivity to Gram-negative pathogens (*E. coli*, *E. coliXen14*, *P. aeruginosaXen41*, *K. pneumoniae*, and *P. mirabilis*) than (*R*)-[^18^F]FMA. Interestingly, (*S*)-[^18^F]FMA showed significantly lower sensitivity in *S. epidermidis* compared with that of (*R*)-[^18^F]FMA. Incorporation in *L. monocytogenes*, *P. aeruginosa*, *A. baumannii*, *S. typhimurium*, and *E. cloacae* was low for both (*S*)- and (*R*)-[^18^F]FMA (Figure 3C). Further studies, including structural elucidation, are needed to understand the origin of this different sensitivities between (*S*)- and (*R*)-[^18^F]FMA in bacterial pathogens.

### Rapid Clearance and Low Background Signals for (*S*)- and (*R*)-[^18^F]FMA Were Observed in Healthy Mice

**Figure 4:**
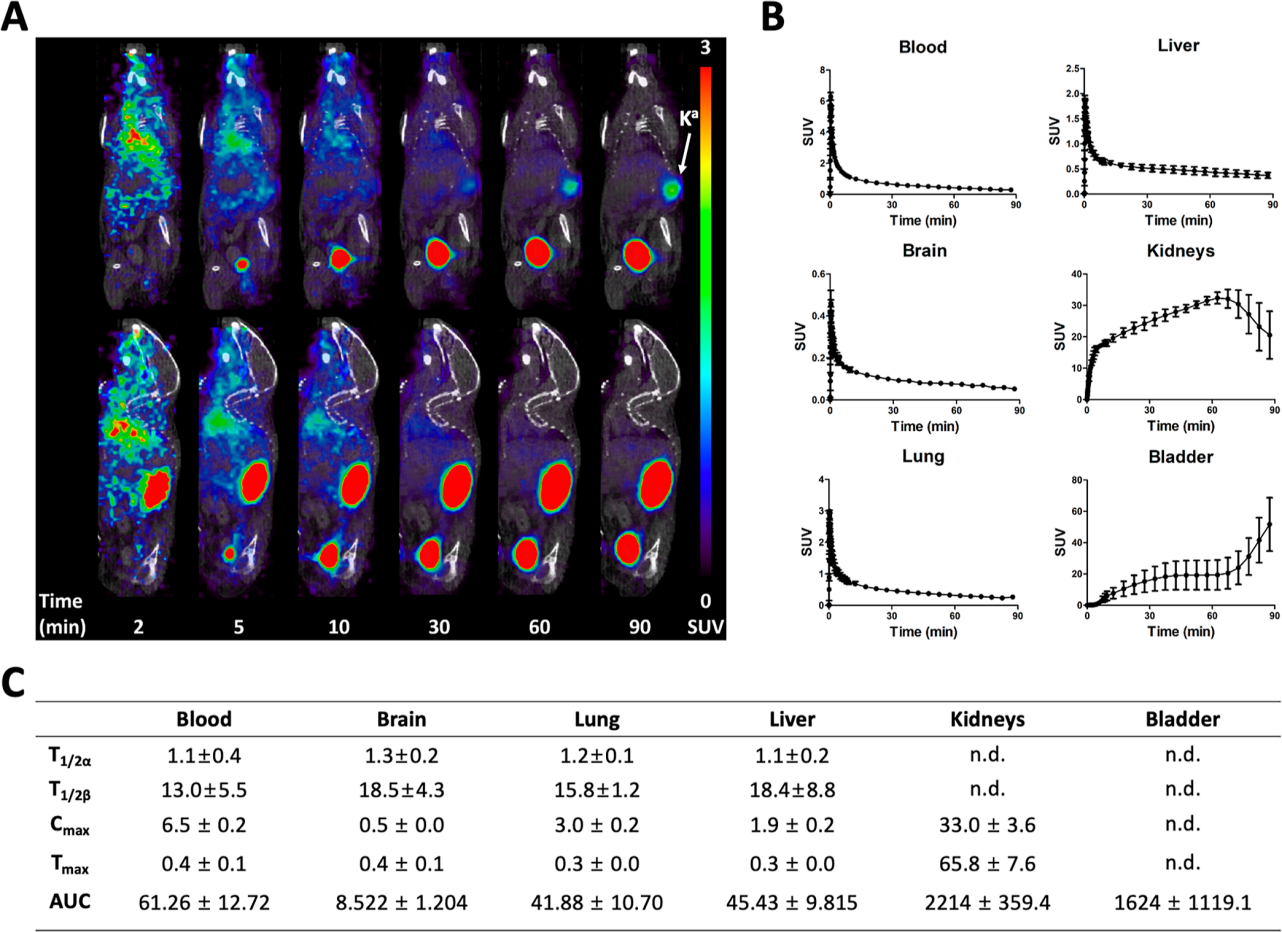
Dynamic μPET/CT imaging analysis of (*S*)-[^18^F]FMA in healthy mice (*n* = 4). (A) Representative time-course μPET/CT imaging of (*S*)-[^18^F]FMA in a healthy mouse. (B) Time-activity curves of (*S*)-[^18^F]FMA in organs of healthy mice. (C) Kinetic parameters of (*S*)-[^18^F]FMA in healthy mice. SUV: standardized uptake value, *T*_1/2_: half-life [min, distribution (α) and elimination (β), respectively], *C*_max_: peak concentration (SUV), *T*_max_: time at *C*_max_ (min), AUC: area under the curve (SUV min), and n.d.: not determined. K^a^: kidney.

We performed preliminary in vivo evaluation of (*S*)- and (*R*)-[^18^F]FMA in healthy mice (CBA/J mice, 9–11 weeks old) to investigate any differences in distribution and stability depending on the stereochemistry of fluorine-18. Following intravenous injection, (*S*)-[^18^F]FMA showed an early peak uptake in the blood, lung, liver, and brain (*T*_max_ < 1 min), followed by rapid washout as shown in time activity curves (TACs) (Figure 4A,B). The highest concentration of (*S*)-[^18^F]FMA was observed early in blood (*C*_max_ = 6.5 ± 0.2 SUV), lung (*C*_max_ = 3.0 ± 0.2 SUV), liver (*C*_max_ = 1.9 ± 0.2 SUV), and brain (*C*_max_ = 0.5 ± 0.0 SUV), followed by rapid clearance (*T*_1/2α_ = 1.1 ± 0.4 min and *T*_1/2β_ = 13.0 ± 5.5 min in blood, *T*_1/2α_ = 1.2 ± 0.1 min and *T*_1/2β_ = 15.8 ± 1.2 min in the lung, *T*_1/2α_ = 1.1 ± 0.2 min and *T*_1/2β_ = 18.4 ± 8.8 min in the liver, and *T*_1/2α_ = 1.3 ± 0.2 min and *T*_1/2β_ = 18.5 ± 4.3 min in the brain, respectively). These results suggested that both (*S*)- and (*R*)-[^18^F]FMA are immediately distributed to the whole-body via systemic and pulmonary circulation after intravenous injection. After rapid washout from those organs, (*S*)-[^18^F]FMA showed a high kidney and bladder uptake. The values of *C*_max_ and AUC of (*S*)-[^18^F]FMA in the kidneys were 15- and 49-fold higher than that of the liver, suggesting that (*S*)-[^18^F]FMA has a dominant urinary excretion pathway rather than biliary excretion (Figure 4C). (*R*)-[^18^F]FMA showed similar distribution/excretion patterns to that of (*S*)-[^18^F]FMA (Supporting Information, Figure S8A–C). The distribution and elimination half-lives were not significantly different between (*S*)- and (*R*)-[^18^F]FMA in the blood, lung, liver, and brain. Both tracers showed high renal accumulation and retention over time, suggesting that the kidneys and bladder are the dose-limiting organs. Ex vivo biodistribution analysis was conducted immediately after the μPET/CT imaging studies. Both tracers showed similar distribution patterns in the organs and tissues, with less than 3% ID g^–1^ of radiotracer in all tissues except for the kidneys. A low bone uptake (<0.4% ID g^–1^) suggested high in vivo stability of both (*S*)- and (*R*)-[^18^F]FMA against ^18^F-defluorination (Supporting Information, Figure S9A,B).

### Both (*S*)- and (*R*)-[^18^F]FMA Could Detect Bacterial Infection in a Murine Myositis Model

**Figure 5:**
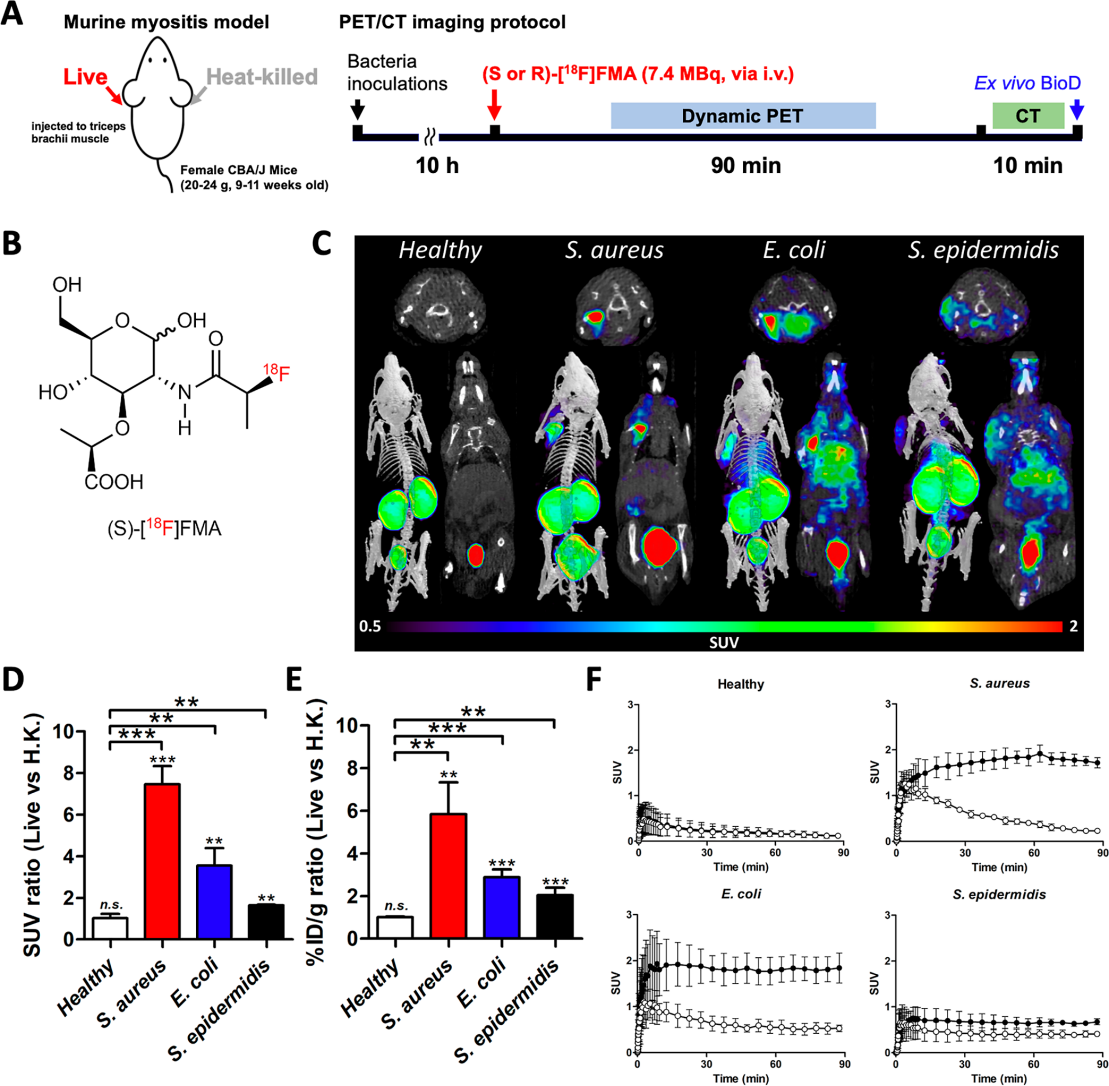
Dynamic μPET/CT imaging analysis of (*S*)-[^18^F]FMA in healthy mice and a murine myositis model (*n* = 4 for each). (A) μPET/CT imaging protocol of (*S*)- and (*R*)-[^18^F]FMA in healthy and murine myositis models. (B) Chemical structure of (*S*)-[^18^F]FMA. (C) Representative μPET/CT images (obtained from the last frame of imaging data; 85–90 min) of (*S*)-[^18^F]FMA in mice. (D) μPET ROI-derived SUV ratio (live vs H.K.) in healthy and infected mice (obtained from the last frame of imaging data; 85–90 min). (E) Ex vivo % ID g^–1^ ratio (live vs H.K.) in healthy and infected mice. (F) Time-activity curves of (*S*)-[^18^F]FMA in live (left muscle, black circle) and heat-killed (right muscle, white circle) in healthy and infected mice. n.s.: not significant, **P < 0.01, ***P < 0.001.

**Figure 6:**
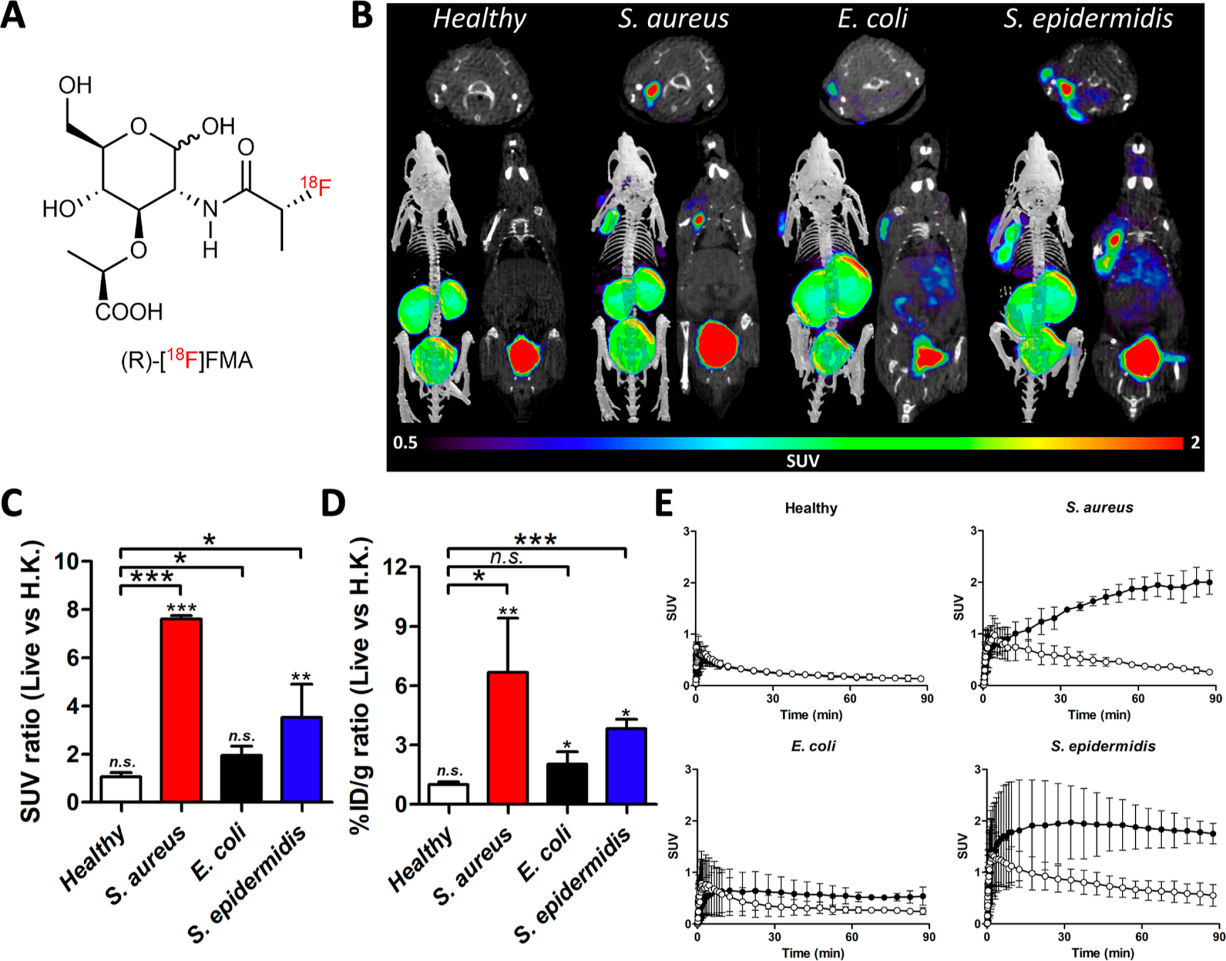
Dynamic μPET/CT imaging analysis of (*R*)-[^18^F]FMA in healthy mice and a murine myositis model (*n* = 4 for each). (A) Chemical structure of (*R*)-[^18^F]FMA. (B) Representative μPET/CT images (obtained from the last frame of imaging data; 85–90 min) of (*R*)-[^18^F]FMA in mice 90 min postinjection. (C) μPET ROI-derived SUV ratio (live vs H.K.) in healthy and infected mice (obtained from the last frame of imaging data; 85–90 min). (D) Ex vivo % ID g^–1^ ratio (live vs H.K.) in healthy and infected mice. (E) Time-activity curves of (*R*)-[^18^F]FMA in live (left muscle, black circle) and heat-killed (right muscle, white circle) in healthy and infected mice. n.s.: not significant, *P < 0.05, **P < 0.01, ***P < 0.001.

Based on in vitro results, we chose three different bacterial pathogens (*S. aureus*, *E. coli*, and *S. epidermidis*) to generate a murine myositis model widely used to screen potential bacteria-specific radiotracers.^6^ Mice were inoculated with live bacteria in the left shoulder and a 10-fold higher concentration than that of heat-killed in the right shoulder, respectively. At 10 h postinjection, dynamic PET scanning was performed immediately 90 min post intravenous injection of (*S*)- and (*R*)-[^18^F]FMA, followed by a 10 min CT scan and ex vivo biodistribution analysis (Figure 5A). μPET/CT images of (*S*)-[^18^F]FMA in both *S. aureus* and *E. coli* infected mice showed a significant tracer uptake at the site of live bacterial inoculation compared with that of heat-killed bacterial inoculation. Even though the accumulation of (*S*)-[^18^F]FMA at the site of live *S. epidermidis* injection was significantly lower than that of both *S. aureus* and *E. coli*, it was visually distinguishable from the heat-killed site, despite the relatively high background of (*S*)-[^18^F]FMA in this model (Figure 5B,C). As shown in the TAC, (*S*)-[^18^F]FMA rapidly accumulated in infected and inflamed muscle at early time points with rapid tracer clearance from inflamed muscle and retention in infected muscle over time. The calculated AUC value from the TAC was higher in infected muscles (146.5 ± 16.4, 157.2 ± 29.3, and 57.5 ± 12.3 for *S. aureus*, *E. coli*, and *S. epidermidis*, respectively) than those of inflamed muscles (50.4 ± 1.5, 56.8 ± 14.8, and 37.6 ± 12.4 for *S. aureus*, *E. coli*, and *S. epidermidis*, respectively). The AUC ratio between infected- and inflamed muscle was 2.91 ± 0.37 (*P* < 0.0001; *S. aureus*-infected), 2.85 ± 0.61 (*P* = 0.0061; *E. coli*-infected), 1.57 ± 0.18 (*P* = 0.1205; *S. epidermidis*-infected), and 1.04 ± 0.17 (*P* = 0.8147; healthy control). SUV ratios between infected and inflamed sites at 90 min postinjection of (*S*)-[^18^F]FMA were significantly higher (7.47 ± 0.87; *P* < 0.0001, 3.55 ± 0.84; *P* = 0.0024, and 1.65 ± 0.02; and *P* = 0.0039 in *S. aureus*, *E. coli*, and *S. epidermidis*, respectively) than that of healthy mice (1.02 ± 0.21; *P* = 0.9109). These data were also supported by % ID g^–1^ ratios (1.00 ± 0.17; n.s. in healthy control, 5.84 ± 1.48; *P* = 0.0075 in *S. aureus*, 2.89 ± 0.37; *P* < 0.0001 in *E. coli*, 2.04 ± 0.35; and *P* = 0.0004 in *S. epidermidis*), which were obtained from ex vivo tissue analysis (Figure 5D–F).

(*R*)-[^18^F]FMA was also robustly accumulated at the site of live *S. aureus* injection but not in that of heat-killed bacterial injection (Figure 6A,B). (*R*)-[^18^F]FMA showed a slightly higher in vitro uptake in *S. aureus* than (*S*)-[^18^F]FMA, but there was no significant difference in accumulation in vivo and ex vivo (in vivo: 1.72 ± 0.11 SUV for (*S*)-[^18^F]FMA, 1.98 ± 0.28 SUV for (*R*)-[^18^F]FMA; *P* = 0.1380. Ex vivo: 2.11 ± 0.44% ID g^–1^ for (*S*)-[^18^F]FMA, 2.55 ± 0.14% ID g^–1^ for (*R*)-[^18^F]FMA; *P* = 0.4408). Unlike (*S*)-[^18^F]FMA, (*R*)-[^18^F]FMA showed high accumulation at the site of live *S. epidermidis* inoculation (SUV: 0.71 ± 0.00 vs 1.80 ± 0.25 for (*S*)- and (*R*)-[^18^F]FMA, respectively; *P* = 0.0014), but low in that of *E. coli* (SUV: 1.67 ± 0.20 vs 0.58 ± 0.23 for (*S*)- and (*R*)-[^18^F]FMA, respectively; *P* = 0.0037), which might be expected based on our in vitro findings. The accumulated ratios (infected versus inflamed sites) of (*R*)-[^18^F]FMA were well matched between PET-derived data (1.06 ± 0.18; n.s. in healthy control, 7.60 ± 0.14; *P* = 0.005 in *S. aureus*, 1.94 ± 0.38; *P* = 0.0603 in *E. coli*, 3.52 ± 1.38; and *P* = 0.0020 in *S. epidermidis*) and ex vivo analysis (1.00 ± 0.12; *P* = 0.9712 in healthy control, 6.67 ± 2.73; *P* = 0.0043 in *S. aureus*, 2.02 ± 0.63; *P* = 0.0332 in *E. coli*, 3.82 ± 0.49; and *P* = 0.0121 in *S. epidermidis*). As shown in the TAC, (*R*)-[^18^F]FMA showed a similar accumulation/washout pattern between infected and inflamed muscle versus that of (*S*)-[^18^F]FMA. The AUC ratio of (*R*)-[^18^F]FMA between infected- and inflamed muscle was 3.11 ± 0.77 (*P* = 0.0003; *S. aureus*-infected), 1.64 ± 0.24 (*P* = 0.2222; *E. coli*-infected), 2.31 ± 0.76 (*P* = 0.0387; *S. epidermidis*-infected), and 0.93 ± 0.07 (*P* = 0.3780; healthy control) (Figure 6C–E). Furthermore, we investigated why tracer performance in vivo did not always correlate with in vitro results. For example, (*S*)-[^18^F]FMA and (*R*)-[^18^F]FMA showed a low uptake in vitro in *S. epidermidis* and *E. coli* but were able to detect these organisms in vivo. The amount of nonspecific binding of both tracers to the filter membrane (as a control; 0.05 ± 0.02% of both tracers were retained) was measured to clarify the specificity of in vitro uptake values (Supporting Information Figure S10A,B). There were significant differences between the control and *S. epidermidis* (*P* < 0.0001) and (*R*)-[^18^F]FMA in *E. coli* (*P* < 0.0001), respectively. These results indicate low but existent uptake of (*S*)-[^18^F]FMA by *S. epidermidis* and (*R*)-[^18^F]FMA by *E. coli* explaining why these tracer/pathogen combinations demonstrated signals in vivo.

**Figure 7:**
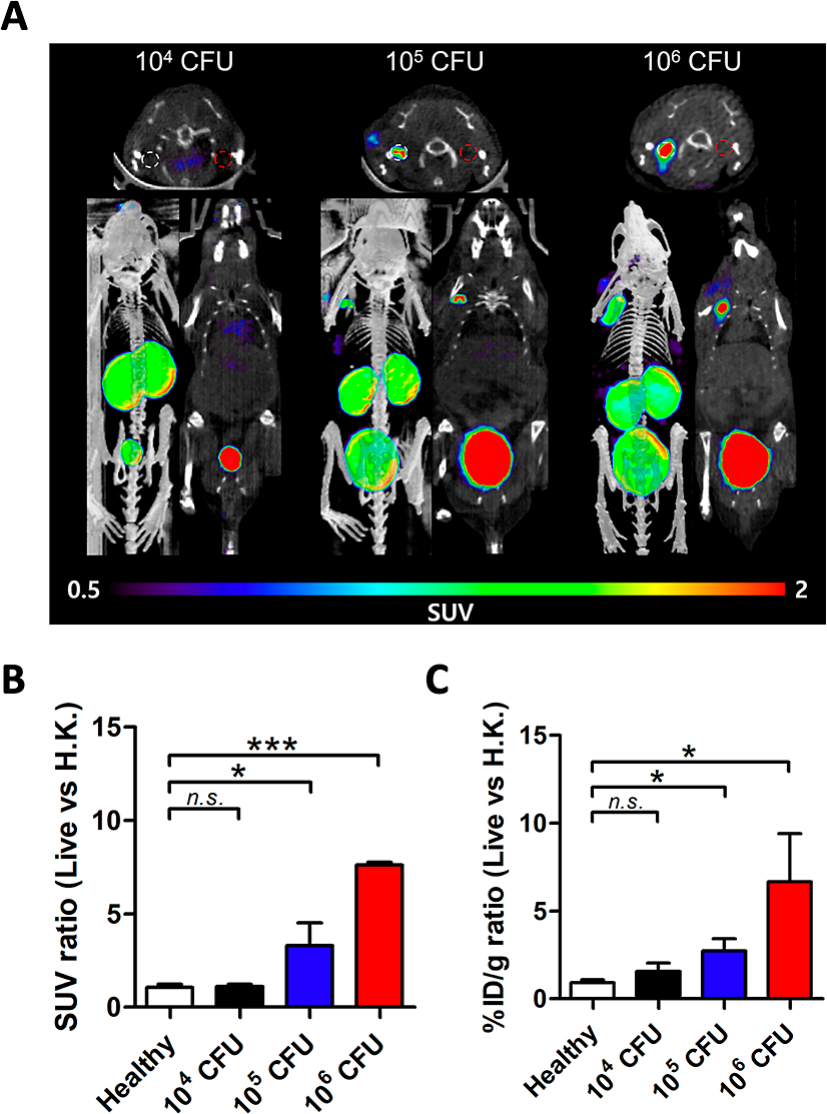
Detection of *S. aureus* in vivo using (*R*)-[^18^F]FMA with variable cfus. (A) Representative μPET/CT images (obtained from the last frame of imaging data; 85–90 min) of (*R*)-[^18^F]FMA in mice 90 min postinjection. Cohorts of mice (*n* = 4 for each) were inoculated with either 10^4^, 10^5^, and 10^6^ cfus *S. aureus* in the left muscle (white dash circle) and 10-fold higher concentration of corresponding heat-killed in the right muscle (red dash circle), respectively. (B) μPET ROI-derived SUV ratio (live vs H.K.) in healthy and infected mice (obtained from the last frame of imaging data; 85–90 min). (C) Ex vivo % ID g^–1^ ratio (live vs H.K.) in healthy and infected mice. n.s.: not significant, *P < 0.05, ***P < 0.001.

To investigate the limit of detection with variable cfus of *S. aureus*, mice were inoculated with 10^4^ and 10^5^ cfus of live *S. aureus* in the left shoulder and a 10-fold higher concentration of heat-killed bacteria in the right shoulder and studied using (*R*)-[^18^F]FMA (Figure 7). μPET/CT images of (*R*)-[^18^F]FMA in 10^5^ cfus *S. aureus*-inoculated mice showed a high uptake of tracer at the site of live bacterial inoculation compared with that of heat-killed bacterial inoculation. The ratio of in vivo (SUV) and ex vivo (% ID g^–1^) PET signals in 10^5^ cfus inoculated mice was decreased compared with that calculated for 10^6^ cfus inoculated mice, but differences were still significant (in vivo: 3.30 ± 1.20; *P* = 0.0260, ex vivo: 2.72 ± 0.70; and *P* = 0.0119). In contrast, μPET/CT images of (*R*)-[^18^F]FMA in 10^4^ cfus of *S. aureus* inoculated mice did not provide visible signals without differences between live and heat-killed inoculations (SUV ratio: 1.10 ± 0.13; *P* = 0.6951,% ID g^–1^ ratio: 1.54 ± 0.48; and *P* = 0.0867).

## Discussion

Many differences between mammalian and bacterial metabolism have been leveraged for imaging, including differential sugar/sugar alcohol uptake,^1,22−24^ iron transport,^25,26^ folic acid biosynthesis,^27−30^ and bacterial cell wall construction.^31^ These differences have been investigated to fulfill the following requirements of infection imaging using PET: (1) high specificity for bacterial infection versus sterile inflammation (2) low uptake in mammalian cells and (3) rapid renal clearance in noninfected tissues to obtain a high signal to background. Among the reported methods, [^18^F]FDS has shown great potential for clinical usage because it can be easily prepared using a kit-based synthesis^32^ from the most general radiotracer ([^18^F]FDG) and has outstanding specificity in vivo. [^18^F]FDS is highly sensitive for Gram-negative *Enterobacteriaceae*, with its major limitation detecting Gram-positive bacteria such as *S. aureus*. More recently, [^18^F]FMtl a positron-emitting mannitol analog developed by Simpson et al., demonstrated the sensitivity to several clinically relevant pathogens including *S. aureus*.^22^ At this point, based on the development of [^18^F]FMtl and the radiotracers reported in this article, it appears that the challenge of developing an *S. aureus*-sensitive ^18^F-tracer has finally been met, with additional comparison and human studies which need to determine which new method is most likely to help patients suffering from bacterial infection.

Both (*S*)- and (*R*)-[^18^F]FMA were studied in healthy mice and a well-characterized murine myositis model. In healthy mice, (*S*)- and (*R*)-[^18^F]FMA showed comparable distribution and excretion patterns, while in the murine myositis model, the in vivo performance closely followed in vitro sensitivity. Somewhat surprisingly, the organism sensitivities of (*S*)- and (*R*)-[^18^F]FMA were not the same. Further studies are needed to explain this phenomenon, but the partial selectivity of (*S*)-[^18^F]FMA for Gram-positive bacteria and (*R*)-[^18^F]FMA for Gram-negative bacteria might be potentially useful diagnostically. Other NAM modifications, such as anhydro-*N*-acetylmuramic acid derivatives,^33,34^ might improve performance in human infection imaging by controlling the kinetics in the body, if needed. Especially as a *S. aureus* sensor, the racemic mixture (*rac*-[^18^F]FMA) showed similar sensitivity to *S. aureus* infection in vivo (Supporting Information, Figure S11), suggesting that new [^18^F]NAc chemistries might circumvent the need for diastereomeric resolution and reduce unnecessary steps for tracer formulation.

## Conclusions

We synthesized the ^18^F-labeled *N*-acetyl muramic acid derivatives (*S*)- and (*R*)-[^18^F]FMA via a simple acylation approach from commercially available muramic acid. The method is applicable to numerous biologically relevant *N*-acetyl amino sugars and sugar alcohols, with future PET methodologies focused on both improving mimicry of the native NAc moiety and obviating the need for enantiomeric/diastereomeric resolution. Despite these limitations, (*S*)- and (*R*)-[^18^F]FMA both showed excellent performance both in vitro and in vivo with marked accumulation by several clinically relevant pathogens including *S. aureus*. The differences in (*S*)- and (*R*)-[^18^F]FMA by several pathogens highlight the subtle structural requirements of peptidoglycan biosynthesis, with the origin of this disparity being evaluated in future studies.

## Supporting Information Available

Syntheses and several imaging studies not reported in the main text and contents of the material (PDF)
