# Supplementary material for: Imaging the Bacterial Cell Wall Using N-Acetyl Muramic Acid-Derived Positron Emission Tomography Radiotracers
Source: ACS Sens. 2023 Nov 22;8(12):4554–65. doi: 10.1021/acssensors.3c01477 (PMC10749472; doi:10.1021/acssensors.3c01477)
Supplement: Supplementary file 1 — se3c01477_si_001.pdf [file se3c01477_si_001.pdf]

Supplementary Information

Imaging the bacterial cell wall using N-acetyl muramic acid-derived positron emission tomography radiotracers

Sang Hee Lee[1], Jung Min Kim[1], Marina López-Álvarez[1], Chao Wang[1], Alexandre M. Sorlin[1], Kondapa Naidu Bobba[1], Priamo A. Pichardo-González[1], Joseph Blecha[1], Youngho Seo[1], Robert R. Flavell[1,2,3], Joanne Engel[4,5], Michael A. Ohliger[1,6*], David M. Wilson[1*]

Affiliations:

[1]Department of Radiology and Biomedical Imaging, University of California, San Francisco, San Francisco, CA 94158, USA.

[2]UCSF Helen Diller Family Comprehensive Cancer Center, University of California, San Francisco, San Francisco, CA 94158, USA.

[3]Department of Pharmaceutical Chemistry, University of California, San Francisco, San Francisco, CA 94158, USA.

[4]Department of Medicine, University of California, San Francisco, San Francisco, CA 94158, USA.

[5]Department of Microbiology and Immunology, University of California, San Francisco, San Francisco, CA 94158, USA.

[6]Department of Radiology, Zuckerberg San Francisco General Hospital, San Francisco, CA 94110, USA.

[*]**Corresponding authors:** David M. Wilson, M.D., Ph.D. (david.m.wilson@ucsf.edu), Michael A. Ohliger, M.D., Ph.D. (michael.ohliger@ucsf.edu)

Contents

A. Chemistry

Scheme S1. Synthesis of (*R,S*)-[$^{19/18}$F]FMA ··· S4

A.1. 4-Nitrophenyl-2-bromopropanoate (**3**) ··· S4

A.2. General synthesis of 4-nitrophenyl-(*R,S*)-2-fluoropropanoate ··· S4

A.2.1. 4-Nitrophenyl-(S)-2-fluoropropanoate ((S)-5) ··· S4

A.2.2. 4-Nitrophenyl-(R)-2-fluoropropanoate ((R)-5) ··· S5

A.3. General synthesis of (2S)-2-(((3R,4R,5S,6R)-3-((R,S)-2-fluoropropanamido)-2,5-dihydroxy-6-(hydroxy methyl)tetrahydro-2H-pyran-4-yl)oxy)propanoic acid ··· S5

A.3.1. (2S)-2-(((3R,4R,5S,6R)-3-((S)-2-fluoropropanamido)-2,5-dihydroxy-6-(hydroxymethyl)tetrahydro-2H-pyran-4-yl)oxy)propanoic acid ((S)-FMA) ··· S5

A.3.2. (2S)-2-(((3R,4R,5S,6R)-3-((R)-2-fluoropropanamido)-2,5-dihydroxy-6-(hydroxymethyl)tetrahydro-2H-pyran-4-yl)oxy)propanoic acid ((R)-FMA) ··· S5

Scheme S2. Synthesis of (S)-NOTsP (8) ··· S6

A.4. (S)-4-Nitrophenyl 2-(tosyloxy)propanoate (8) ··· S6

B. ^{1}H and ^{13}C NMR spectra ··· S7

C. Supplemental Figures and Table

Fig. S1 HPLC profile of [^{18}F]NFP ··· S17

Fig. S2 Representative radio-TLC profiles of (R,S)-[^{18}F]FMA in saline, mouse serum, and human serum at 37°C for 0, 15, 30, 60, and 90 min, respectively ··· S18

Fig. S3 Radiosynthesis of (R)-[^{18}F]FMA from (S)-NOTsP (8) ··· S19

Fig. S4 Analytical HPLC profile of [^{18}F]FPA co-injected with cold standard ··· S20

Fig. S5 Analytical HPLC profile of (*R,S*)-[^{18}F]FMA co-injected with cold standards at different time ··· S21

Fig. S6 Heat-promoted mutarotation analysis of (*R,S*)-[^{18}F]FMA ··· S22

Fig. S7 *In vitro* analyses of (*R,S*)-[^{18}F]FMA in *S. aureus* before and after α-β equilibration ··· S23

Fig. S8 Dynamic PET/CT imaging analysis of (R)-[^{18}F]FMA in healthy mice ··· S24

Fig. S9 *Ex vivo* biodistribution of (*S*)-[^{18}F]FMA (A) and (*R*)-[^{18}F]FMA (B) in healthy mice ··· S25

Fig. S10 *In vitro* cellular uptake of (*S*)- and (*R*)-[^{18}F]FMA compared with the control ··· S26

Fig. S11 Comparison of *in vitro* and *in vivo* detection sensitivity of the tracers in *S. aureus* ··· S27

Fig. S12 Dynamic PET/CT imaging analysis of (*R,S*)-[^{18}F]FMA in murine myositis model ··· S28

Fig. S13 *Ex vivo* biodistribution of (*R,S*)-[^{18}F]FMA in murine myositis model ··· S29

Table. S1 Bacteria strains and growth conditions used for this study ········· S30

D. Reference ········· S31

A. Chemistry

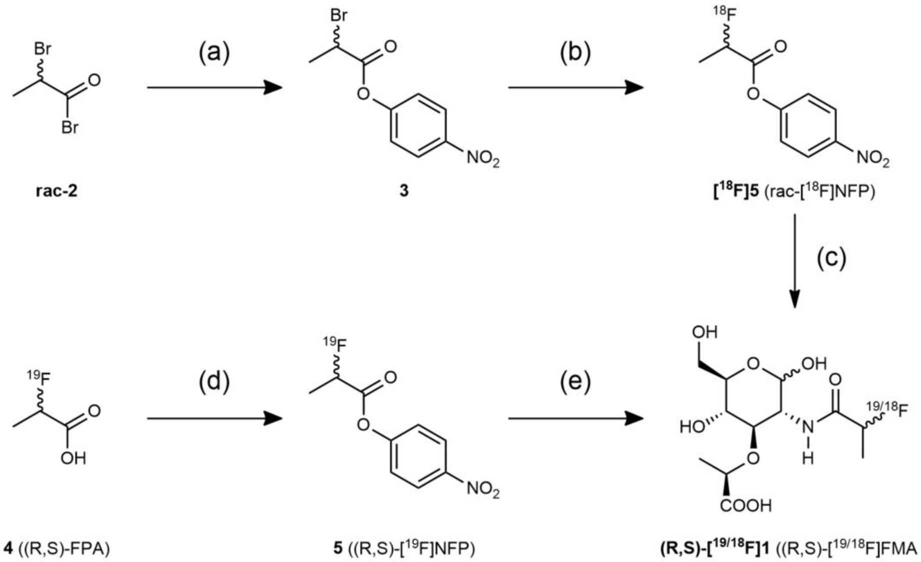

Scheme S1. Synthesis of (R,S)-[$^{19/18}$F]FMA. Reagent and conditions: (a) triethylamine (1.0 eq), 4-nitrophenol (1.03 eq), DCM, 0 ^{o}C to r.t., 2h. (b) precursor (8 mg, 29.2 μmol), $^{18}F^{-}$, Cs_2CO_3 (2.2 mg, 6.8 μmol), 80% tBuOH/MeCN (0.5 mL), 110 ^{o}C, 10 min. (c) α-muramic acid (0.25 mg, 1.0 μmol), 0.1% TEA/DMSO (100 μL), 60 ^{o}C, 10 min. (d) 4-(dimethylamino)pyridine (0.1 eq), *N,N'*-dicyclohexylcarbodiimide (1.04 eq), 4-nitrophenol (1.04 eq), DCM, 0 ^{o}C to r.t., 2h. (e) α-muramic acid (0.8 eq), triethylamine (0.72 eq), DMSO, 60 ^{o}C, 20 min.

A.1. 4-Nitrophenyl-2-bromopropanoate (3)

To a stirred solution of 2-bromopropanoyl bromide (2.5 g, 11.6 mmol) dissolved in DCM (20 mL), triethylamine (1.6 mL, 11.6 mmol) and 4-nitrophenol (1.65 g, 11.9 mmol) dissolved in DCM (10 mL) was added dropwise at 0 ^{o}C under nitrogen. The mixture was stirred at room temperature for 2 h. Once 4-nitrophenol has been consumed, monitored by TLC, the solvent was removed under reduced pressure. The concentrated mixture was purified by silica gel column chromatography (gradient from 1:6 to 1:2 of n-hexane:ethyl acetate) to afford the desired product as a white solid (2.1g, 66%). ^{1}H NMR (400 MHz, $CDCl_3$) δ 8.30 (d, *J* = 9.2 Hz, 2H), 7.33 (d, *J* = 9.2 Hz, 2H), 4.61 (q, *J* = 6.8 Hz, 1H), 1.96 (d, *J* =7.2 Hz, 3H); ^{13}C NMR (100 MHz, $CDCl_3$) δ 168.0, 155.2, 145.8, 125.5, 122.2, 39.1, 21.4. HRMS (EI-Pos) m/z [M] calcd for $C_9H_8BrNO_4$ 272.9637, found 272.9631.

A.2. General synthesis of 4-nitrophenyl-(R,S)-2-fluoropropanoate

(R,S)-2-fluoropropanoic acid (250 mg, 2.7 mmol) was added to the mixture of 4-(dimethylamino)pyridine (30 mg, 0.25 mmol) and 4-Nitrophenol (390 mg, 2.8 mmol) in 10 mL of DCM and stirred at 0 ^{o}C for 10 min under nitrogen. *N,N'*-dicyclohexylcarbodiimide (578 mg, 2.8 mmol) in 10 mL of DCM was added slowly to the mixture at 0 ^{o}C, then stirred at room temperature for 2 h. The resulting mixture was further diluted by 10 mL of DCM, then washed with water (twice) and brine, followed by dried over with anhydrous sodium sulfate and concentrated under the reduced pressure. The crude mixture was purified by silica gel column chromatography (gradient from 1:6 to 1:2 of n-hexane:ethyl acetate) to afford the desired product.

A.2.1. 4-Nitrophenyl (S)-2-fluoropropanoate ((S)-5)

White solid (294 mg, 51%). ^1H NMR (400 MHz, $CDCl_3$) δ 8.30 (d, *J* = 9.2 Hz, 2H), 7.35 (d, *J* = 9.2 Hz, 2H), 5.28 (dq, *J* = 48.4 and 7.2 Hz, 1H), 1.77 (dd, *J* = 23.4 and 7.2 Hz, 3H); ^{13}C NMR (100 MHz, $CDCl_3$) δ 168.1 (d, *J* = 24.6 Hz, CO-CHF), 154.7, 145.9, 125.5, 122.3, 85.5 (d, *J* = 183.5 Hz, CF), 18.4 (d, *J* = 22.3 Hz, CH_3-CF); ^{19}F NMR (376 MHz, $CDCl_3$) δ -184.57 (sext, *J* = 23.7 Hz). HRMS (EI-Pos) m/z [M] calcd for $C_9H_8FNO_4$ 213.0434, found 213.0434.

A.2.2. 4-Nitrophenyl (R)-2-fluoropropanoate ((R)-5)

White solid (325 mg, 56%). ^1H NMR (400 MHz, $CDCl_3$) δ 8.30 (d, *J* = 9.2 Hz, 2H), 7.34 (d, *J* = 9.2 Hz, 2H), 5.28 (dq, *J* = 48.0 and 6.8 Hz, 1H), 1.77 (dd, *J* = 23.6 and 6.8 Hz, 3H); ^{13}C NMR (100 MHz, $CDCl_3$) δ 168.1 (d, *J* = 24.6 Hz, CO-CHF), 154.7, 145.9, 125.5, 122.3, 85.5 (d, *J* = 183.5 Hz, CF), 18.4 (d, *J* = 22.3 Hz, CH_3-CF); ^{19}F NMR (376 MHz, $CDCl_3$) δ -184.55 (sext, *J* = 23.7 Hz). HRMS (EI-Pos) m/z [M] calcd for $C_9H_8FNO_4$ 213.0434, found 213.0437.

A.3. General synthesis of (2S)-2-(((3R,4R,5S,6R)-3-((R,S)-2-fluoropropanamido)-2,5-dihydroxy-6-(hydroxymethyl)tetrahydro-2H-pyran-4-yl)oxy)propanoic acid

To a stirred solution of muramic acid (20 mg, 80 μmol) and triethylamine (7.3 mg, 72 μmol) dissolved in dimethyl sulfoxide (300 μL), 4-nitrophenyl-(R,S)-2-fluoropropanoate (22 mg, 0.1 mmol) was added. The reaction mixture was stirred at 60 ^oC for 20 min. The yellow mixture was diluted with 5 mL of diethyl ether, then extracted by using 1 mL of aqueous 0.1 N HCl (twice). The collected aqueous layer was combined, then purified on the semi-prep HPLC system (Phenomenex, Luna 10 μm C18 column, 250 × 10 mm; 5% EtOH/Water containing 0.1% HCl; λ = 210 nm, flow rate = 4.0 $mLmin^{-1}$).

A.3.1. (2S)-2-(((3R,4R,5S,6R)-3-((S)-2-fluoropropanamido)-2,5-dihydroxy-6-(hydroxymethyl)tetra hydro-2H-pyran-4-yl)oxy)propanoic acid ((S)-FMA)

White solid (8.4 mg, 32.3 %), T_R = 15 min; ^1H NMR (400 MHz, D_2O) δ 5.36 (d, *J* = 3.2 Hz, 1H, H-1α), 5.13 (dd, *J* = 48.2 and 6.8 Hz, 1H, H-CF α), 5.08 (dd, *J* = 41.2 and 7.2 Hz, 1H, H-CF β), 4.60 (q, *J* = 6.8 Hz, 1H, CH-CH3 muramic acid α), 4.41 (q, *J* = 6.8 Hz, 1H, CH_3 muramic acid β), 3.97-3.90 (m, 1H, H-6β), 3.92-3.82 (m, 5H, H-6'β, H-6α, H-6'α, H-5α, H-4α), 3.82-3.75 (m, 1H, H-2α), 3.67-3.60 (m, 2H, H-3α, H-5β), 3.60-3.52 (m, 1H, H-3β), 3.52-3.45 (m, H-2β), 1.49 (dd, *J* = 25.2 and 6.8 Hz, 3H, CH_3-CF α), 1.48 (dd, *J* = 25.2 and 6.8 Hz, 3H, CH_3-CF β), 1.48 (d, *J* = 7.2 Hz, CH_3 muramic acid α), 1.46 (d, *J* = 8.4 Hz, CH_3 muramic acid β); ^{13}C NMR (100 MHz, CD_3OD) δ 176.6, 173.6 (d, *J* = 20.0 Hz, CO-CHF), 91.6, 89.2 (d, *J* = 181.2 Hz, CF), 78.5, 76.5, 73.4, 73.2, 62.3, 55.3, 19.2, 18.9 (d, *J* = 22.0 Hz, CH_3-CF); ^{19}F NMR (376 MHz, D_2O) δ -182.35 (α, sext, *J* = 22.6 Hz), -182.36 (β, sext, *J* = 23.7 Hz). HRMS (ESI-Neg) m/z $[M-H]^-$ calcd for $C_{12}H_{19}FNO_8^-$ 324.1095, found 324.1098.

A.3.2. (2S)-2-(((3R,4R,5S,6R)-3-((R)-2-fluoropropanamido)-2,5-dihydroxy-6-(hydroxymethyl)tetrahydro-2H-pyran-4-yl)oxy)propanoic acid ((R)-FMA)

White solid (8.2 mg, 31.5 %), T_R = 18 min; ^1H NMR (400 MHz, D_2O) δ 5.37 (d, *J* = 3.2 Hz, 1H, H-1α), 5.16 (dd, *J* = 48.2 and 6.8 Hz, 1H, H-CF α), 5.14 (dd, *J* = 41.2 and 7.2 Hz, 1H, H-CF β), 4.61 (q, *J* = 6.8 Hz, 1H, CH-CH_3 muramic acid α), 4.46 (q, *J* = 7.2 Hz, 1H, CH_3 muramic acid β), 3.97-3.90 (m, 1H, H-6β), 3.92-3.82 (m, 5H, H-6'β, H-6α, H-6'α, H-5α, H-4α), 3.82-3.74 (m, 1H, H-2α), 3.72-3.61 (m, 2H, H-3α, H-5β), 3.61-3.53 (m, 1H, H-3β), 3.54-3.47 (m, H-2β), 1.57 (dd, *J* = 25.2 and 6.8 Hz, 3H, CH_3-CF α), 1.56 (dd, *J* = 24.8 and 6.8 Hz, 3H, CH_3-CF β), 1.47 (d, *J* = 7.2 Hz, CH_3 muramic acid α), 1.45 (d, *J* = 8.4 Hz, CH_3 muramic acid β); ^{13}C NMR (100 MHz, CD_3OD)

δ 176.6, 173.5 (d, J = 20.1 Hz, CO-CHF), 91.7, 89.3 (d, J = 181.3 Hz, CF), 78.3, 76.4, 73.5, 73.3, 62.4, 55.3, 19.2, 18.7 (d, J = 22.1 Hz, CH_3-CF); ^{19}F NMR (376 MHz, D_2O) δ -182.65 (α, sext, J = 24.8 Hz), -182.78 (β, sext, J = 24.8 Hz). HRMS (ESI-Neg) m/z $[M-H]^-$ calcd for $C_{12}H_{19}FNO_8^-$ 324.1095, found 324.1098.

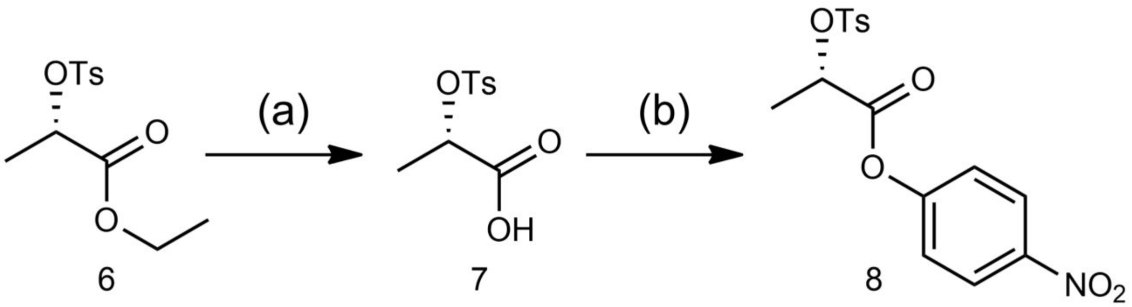

Scheme S2. Synthesis of (S)-NOTsP (**8**). Reagent and conditions: (a) sodium hydroxide (1.2 eq), 40% EtOH/Water, 0 ^{o}C, 5h. (b) 4-(dimethylamino)pyridine (0.1 eq), 4-nitrophenol (1.0 eq), *N,N'*-dicyclohexylcarbodiimide (1.0 eq), DCM, 0 ^{o}C to r.t., 6h.

A.4. (S)-4-Nitrophenyl 2-(tosyloxy)propanoate (8)

(S)-2-(tosyloxy)propanoic acid (**7**) was synthesized according to the literature [1]. To a stirred solution of (S)-ethyl 2-(tosyloxy)propanoate (**6**, 12 g, 44 mmol) dissolved in ethanol (12 mL), a solution of sodium hydroxide (2.1 g, 52.5 mmol) dissolved in water (18 mL) was added slowly at 0 ^{o}C. The reaction mixture was stirred at 0 ^{o}C until compound **6** completely disappeared (5 h), monitored by TLC. The reaction mixture was further diluted by water, acidified with 3 N HCl to pH 4, followed by extraction with dichloromethane (50 mL x 3). The combined organic layer was dried over anhydrous sodium sulfate and concentrated under reduced pressure to afford the compound **7** quantitively (9.8 g, 40 mmol) which was used for the next esterification without further purification.

The compound **7** (9.8 g, 40 mmol), 4-(dimethylamino)pyridine (480 mg, 4 mmol) and 4-Nitrophenol (5.5 g, 40 mmol) was dissolved in DCM (30 mL) and stirred at 0 ^{o}C for 30 min under nitrogen. *N,N'*-dicyclohexylcarbodiimide (8.3 g, 40 mmol) in 25 mL of DCM was added slowly to the mixture at 0 ^{o}C, then stirred at room temperature for 6 h. The resulting mixture was washed with water (60 mL x 2) and brine (60 mL), followed by dried over anhydrous sodium sulfate and concentrated under reduced pressure. The crude mixture was purified by silica gel column chromatography (gradient from 1:8 to 1:1 of n-hexane:ethyl acetate) to afford the compound **8** as a white solid (6.6 g, 45%).; ^{1}H NMR (400 MHz, $CDCl_3$) δ 8.27 (d, J = 9.2 Hz, 2H), 7.85 (d, J = 8.4 Hz, 2H), 7.37 (d, J = 8.4 Hz, 2H), 7.25 (d, J = 8.4 Hz, 2H), 5.13 (q, J = 6.8 Hz, 1H), 2.46 (s, 3H), 1.67 (d, J =7.8 Hz, 3H); ^{13}C NMR (100 MHz, $CDCl_3$) δ 167.3, 154.7, 145.9, 145.7, 133.0, 130.1, 128.2, 125.4, 122.3, 73.6, 21.8, 18.3. HRMS (ESI-Pos) m/z $[M+Na]^+$ calcd for $C_{16}H_{15}NNaNO_7S^{-+}$ 388.0466, found 388.0462.

B. ^{1}H and ^{13}C NMR spectra

B.1.1. 4-Nitrophenyl-2-bromopropanoate ^{1}H NMR (400 MHz, $CDCl_3$)

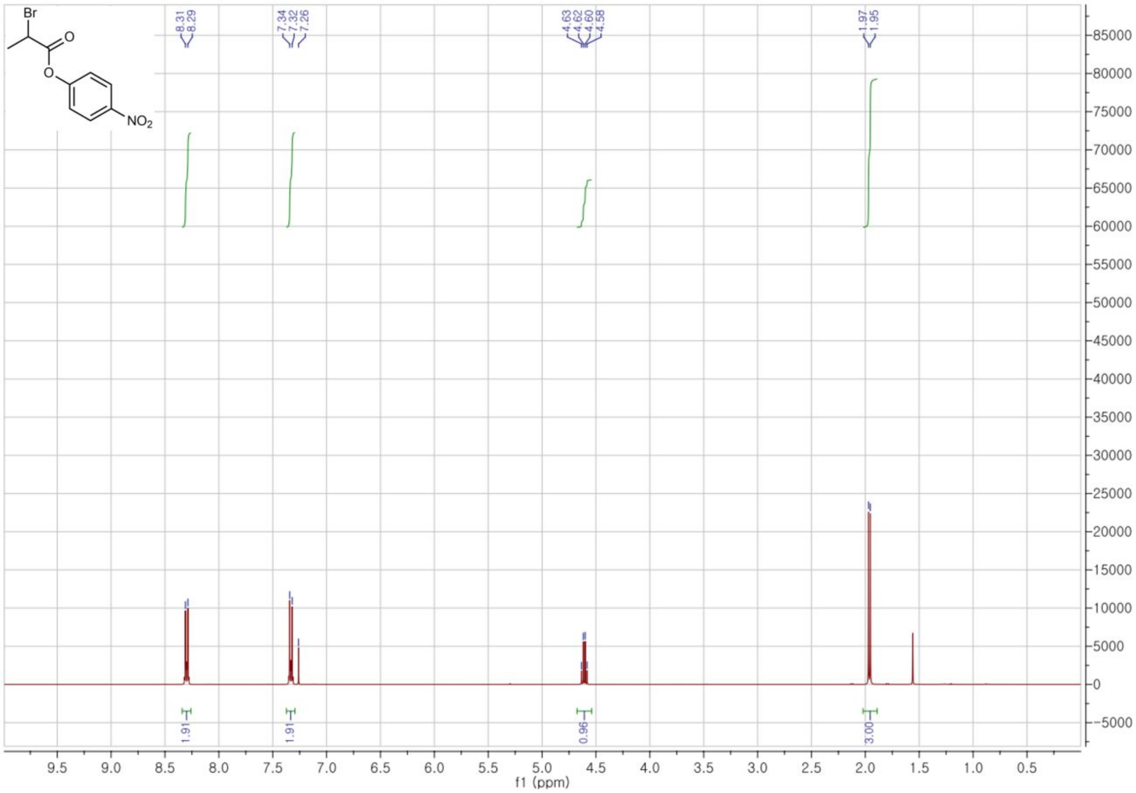

B.1.2. 4-Nitrophenyl-2-bromopropanoate ^{13}C NMR (100 MHz, $CDCl_3$)

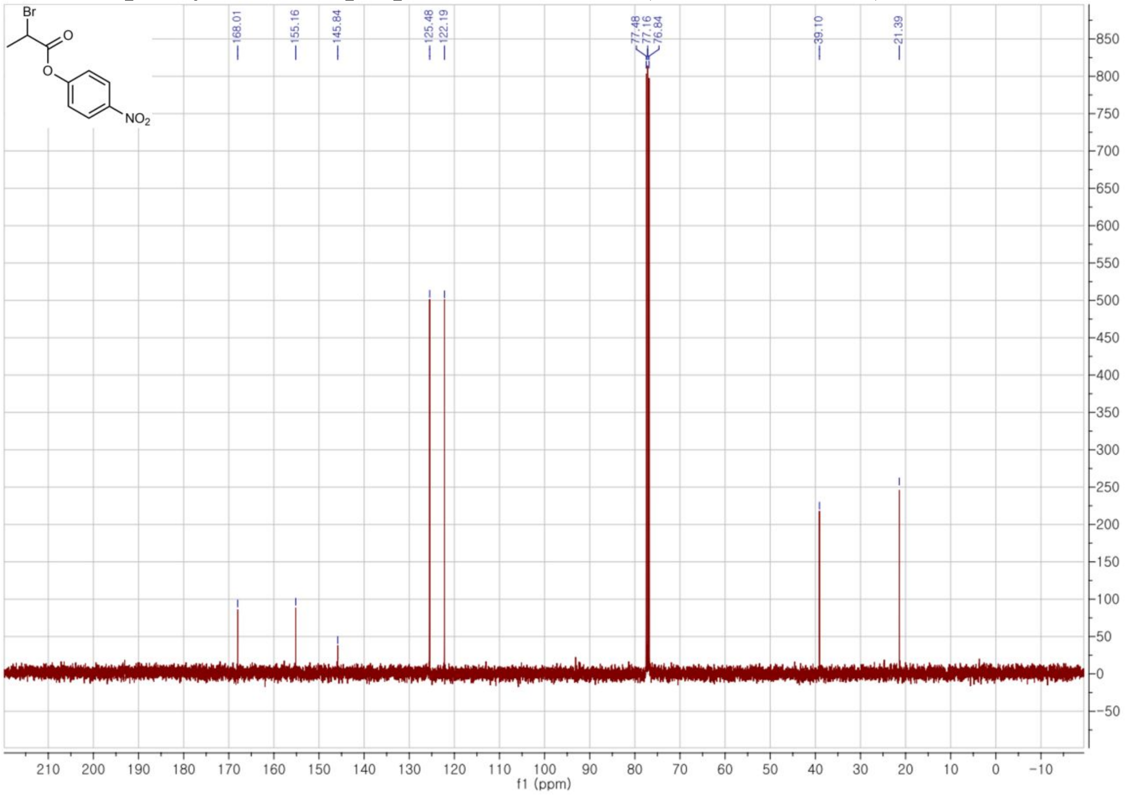

B.2.1. 4-Nitrophenyl-(S)-2-fluoropropanoate ^{1}H NMR (400 MHz, $CDCl_3$)

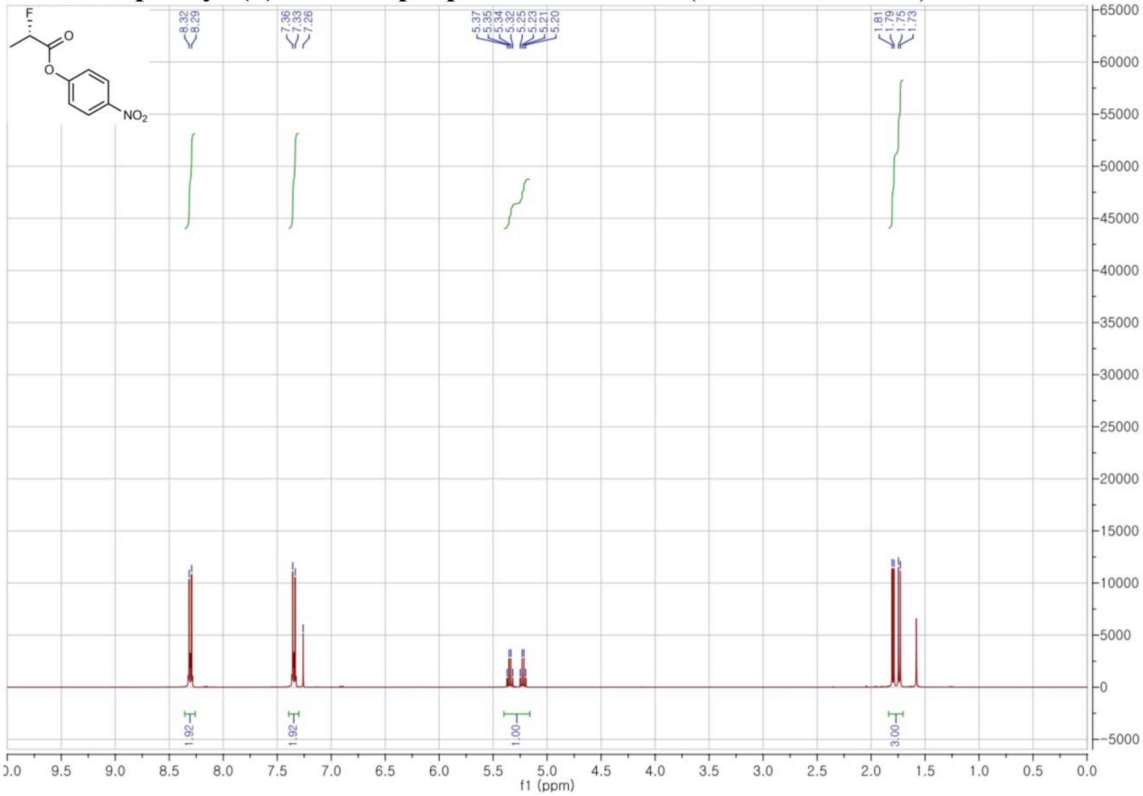

B.2.2. 4-Nitrophenyl-(S)-2-fluoropropanoate ^{13}C NMR (100 MHz, $CDCl_3$)

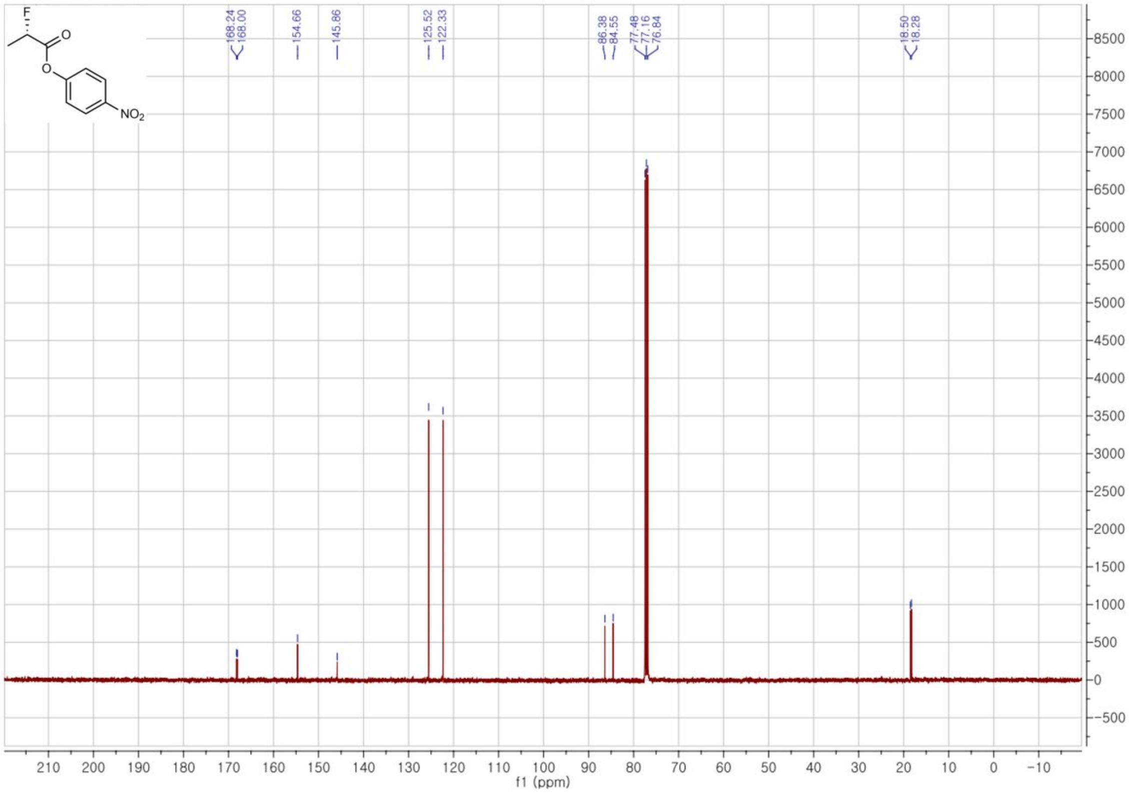

B.2.3. 4-Nitrophenyl-(S)-2-fluoropropanoate ^{19}F NMR (376 MHz, $CDCl_3$)

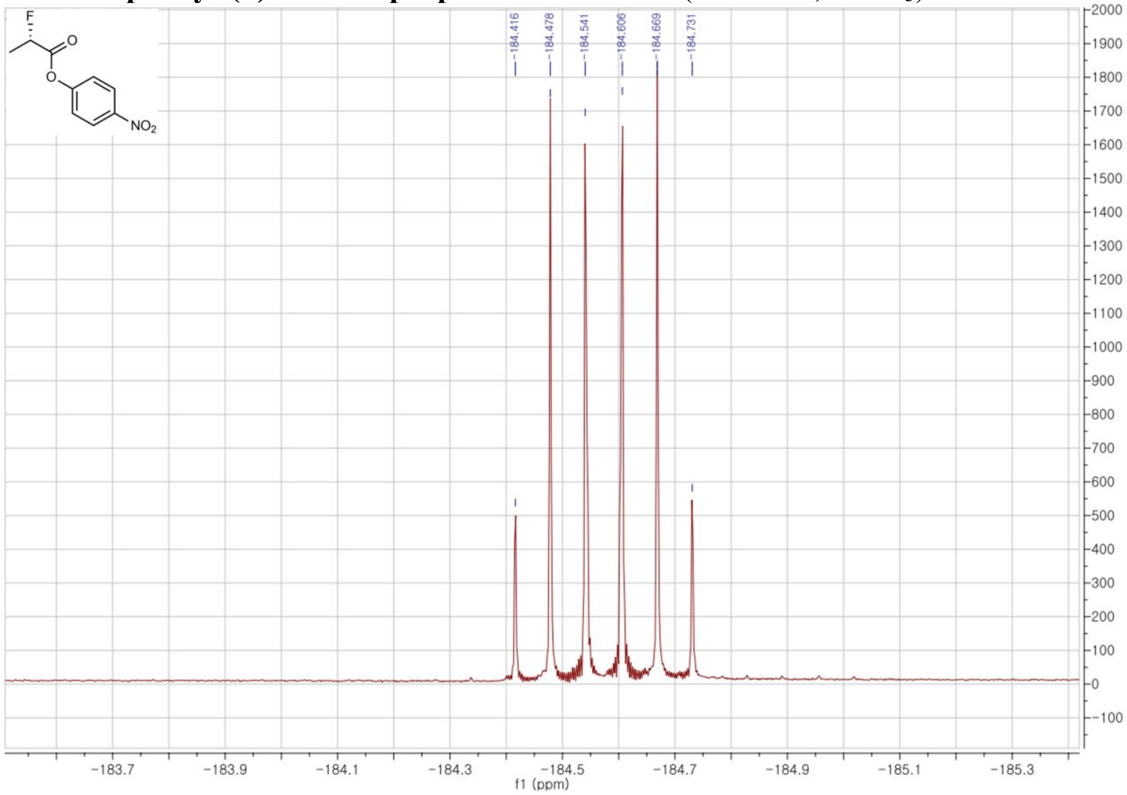

B.3.1. 4-Nitrophenyl-(R)-2-fluoropropanoate ^{1}H NMR (400 MHz, $CDCl_3$)

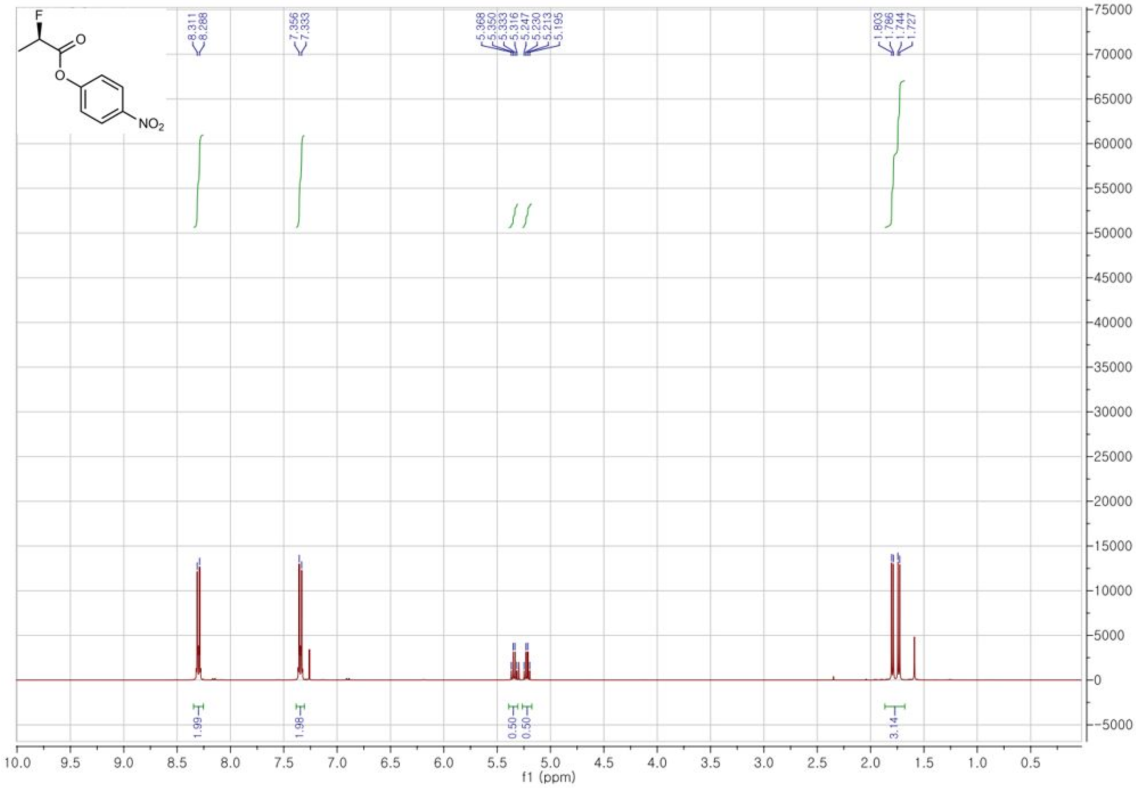

B.3.2. 4-Nitrophenyl-(R)-2-fluoropropanoate ^{13}C NMR (100 MHz, $CDCl_3$)

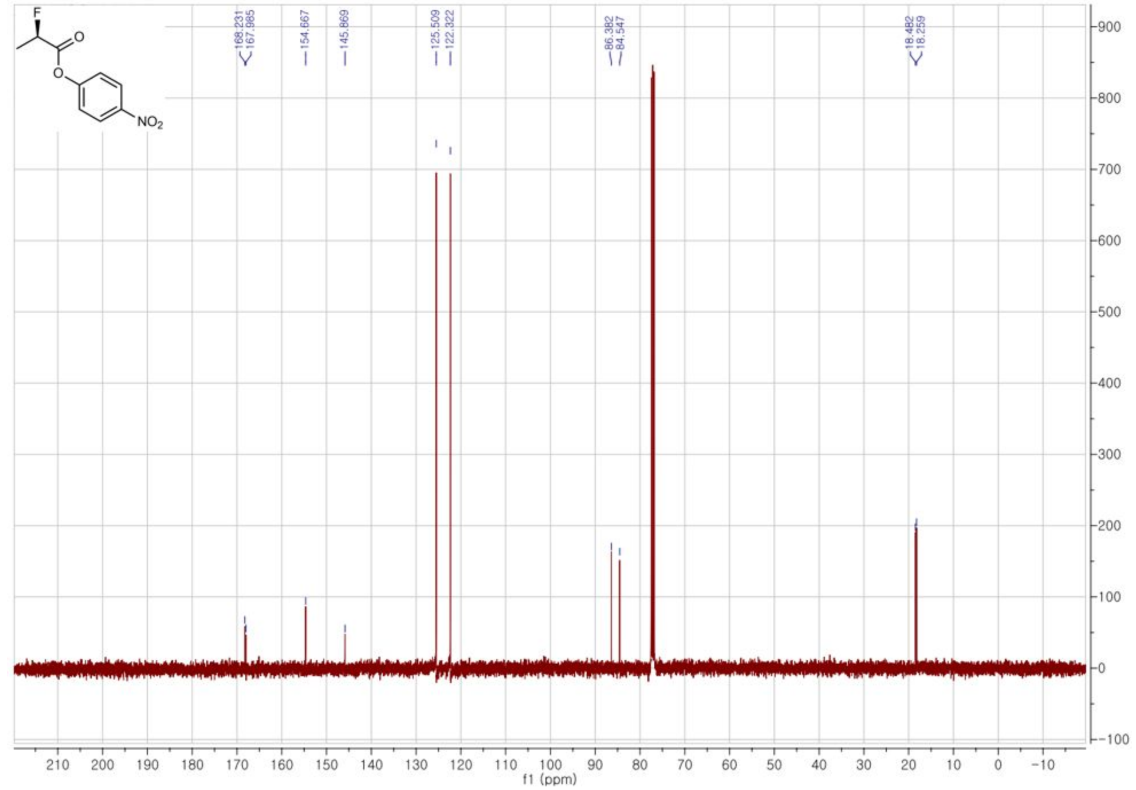

B.3.3. 4-Nitrophenyl-(R)-2-fluoropropanoate ^{19}F NMR (376 MHz, $CDCl_3$)

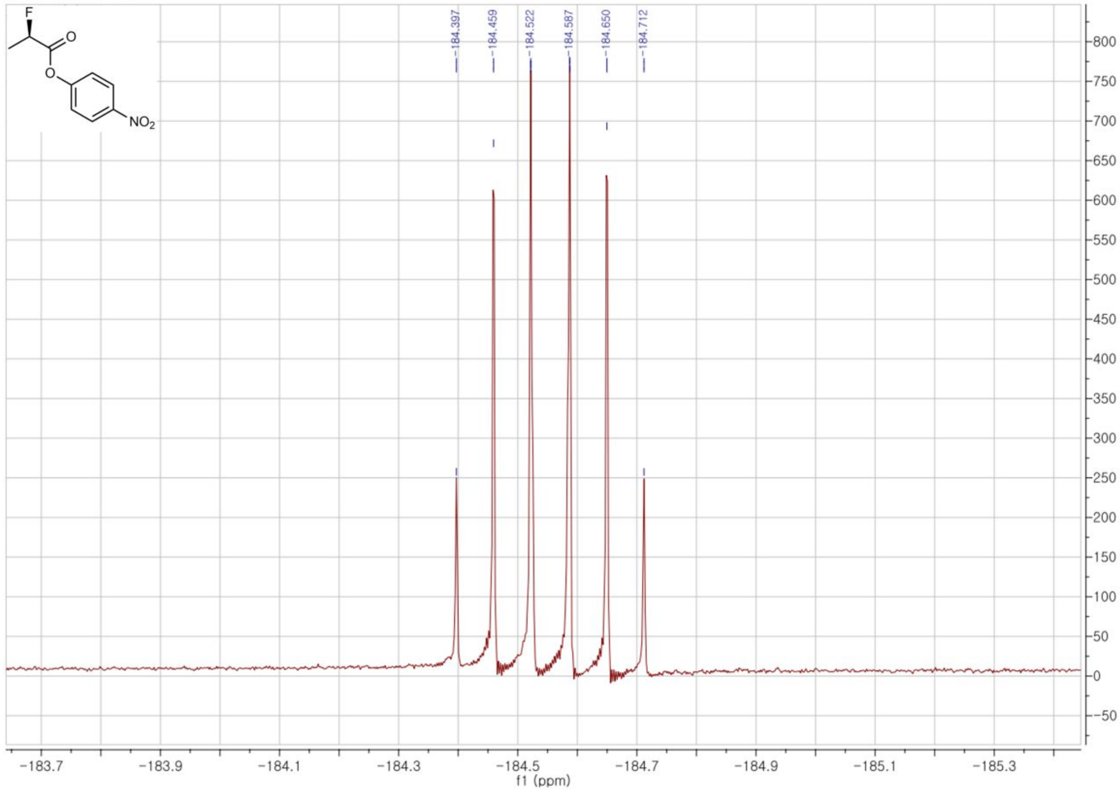

B.4.1. (S)-FMA ^{1}H NMR (400 MHz, D_2O)

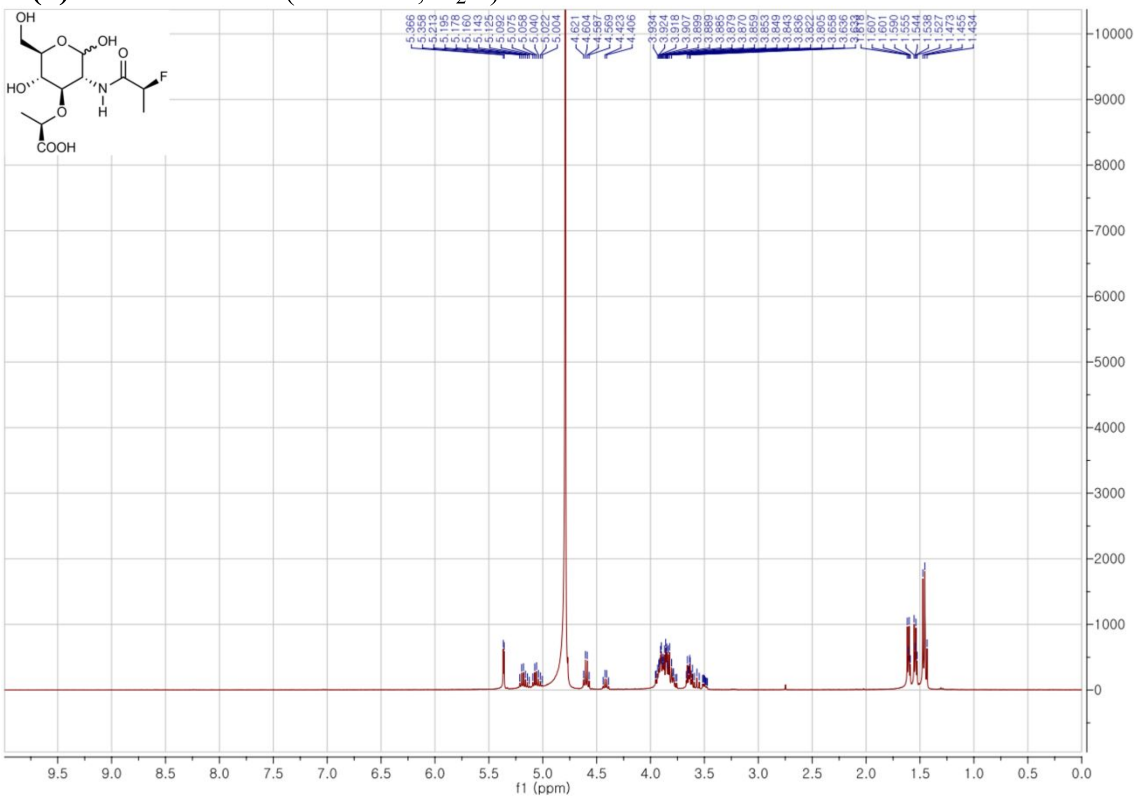

B.4.2. (S)-FMA ^{13}C NMR (100 MHz, CD_3OD)

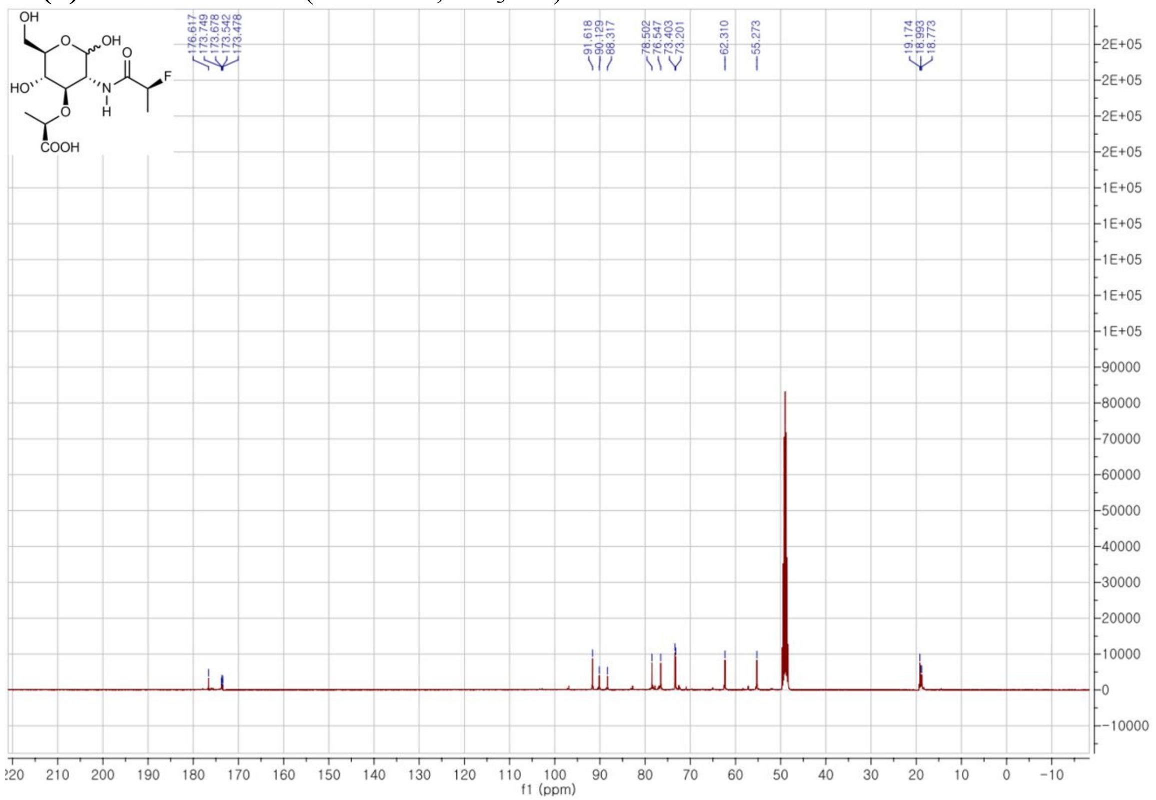

B.4.3. (S)-FMA ^{19}F NMR (376 MHz, D_2O)

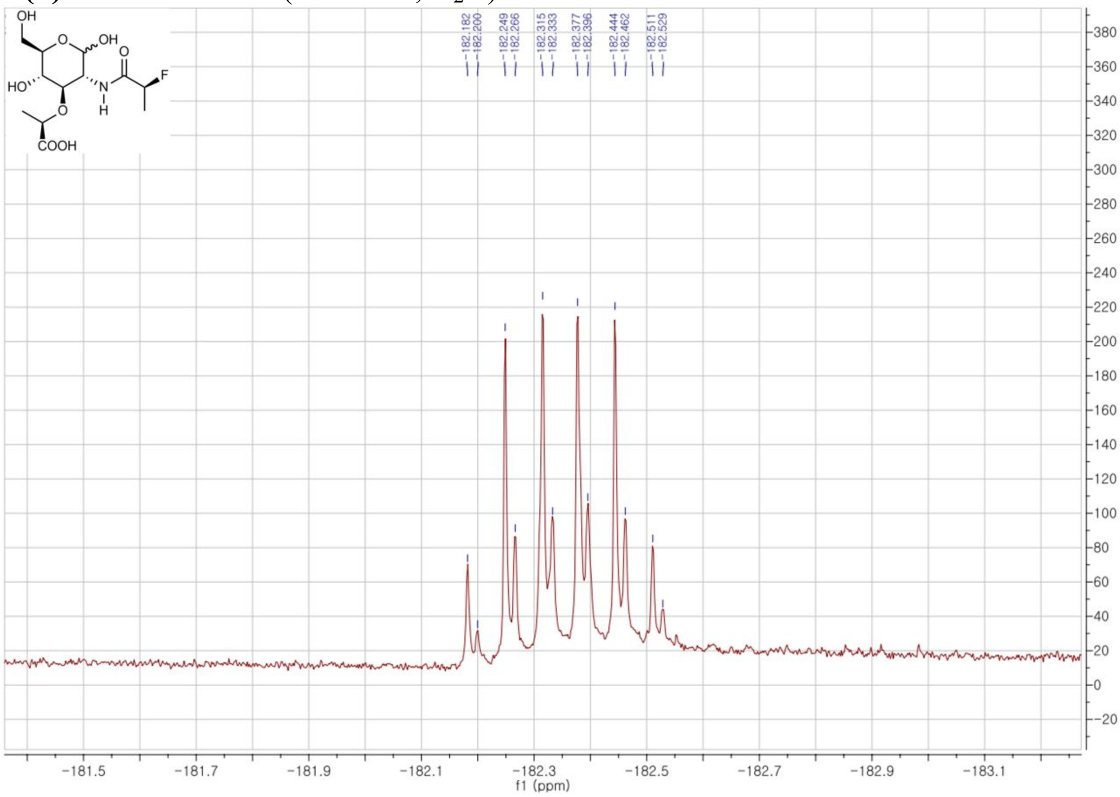

B.5.1. (R)-FMA ^{1}H NMR (400 MHz, D_2O)

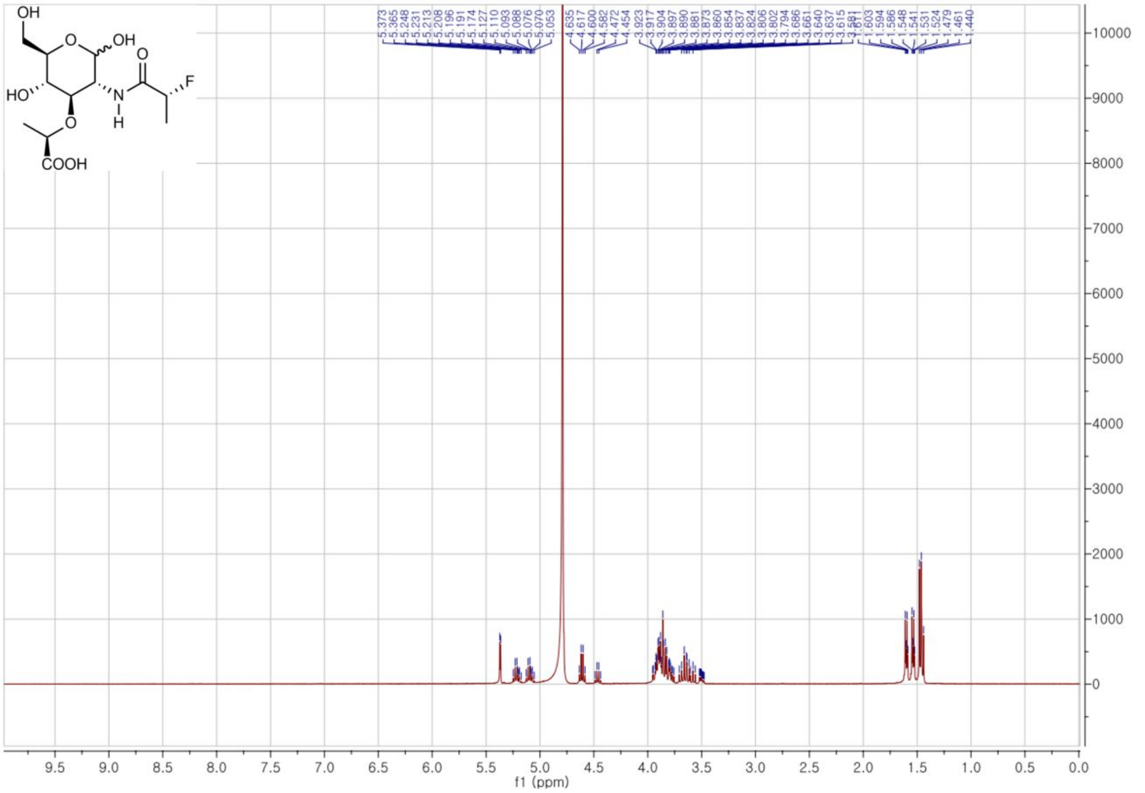

B.5.2. (R)-FMA ^{13}C NMR (100 MHz, CD_3OD)

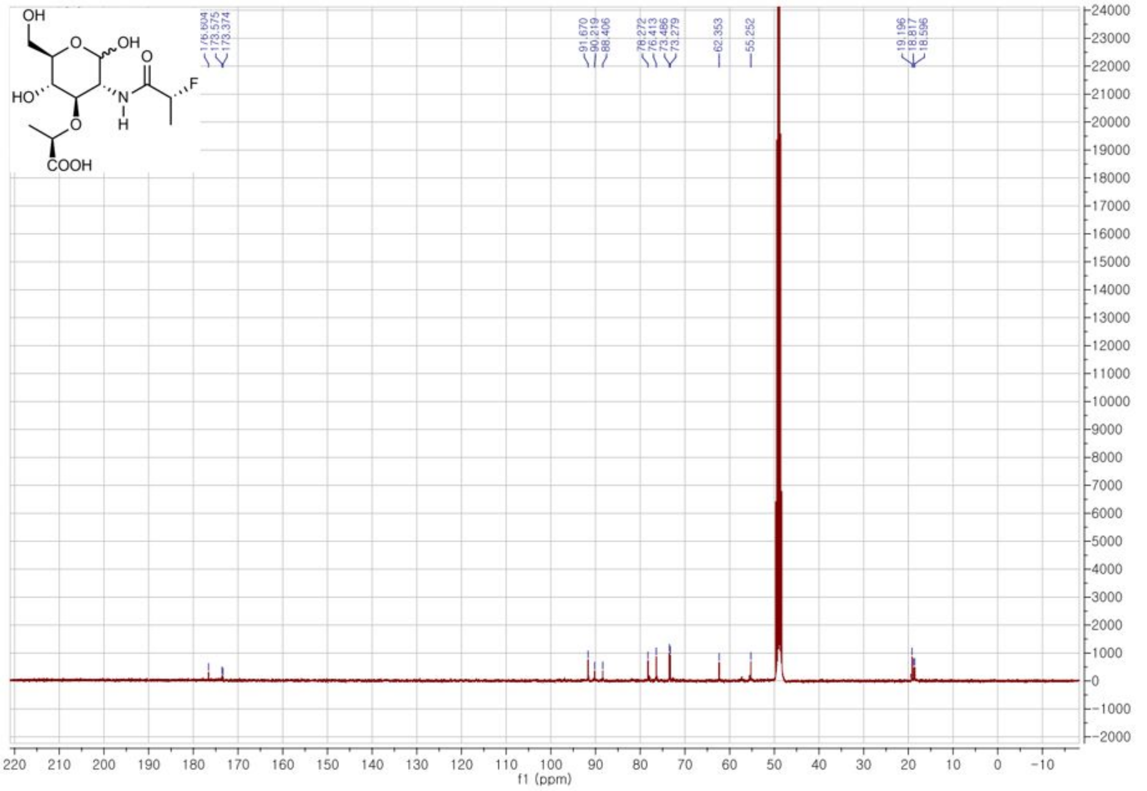

B.5.3. (R)-FMA ^{19}F NMR (376 MHz, D_2O)

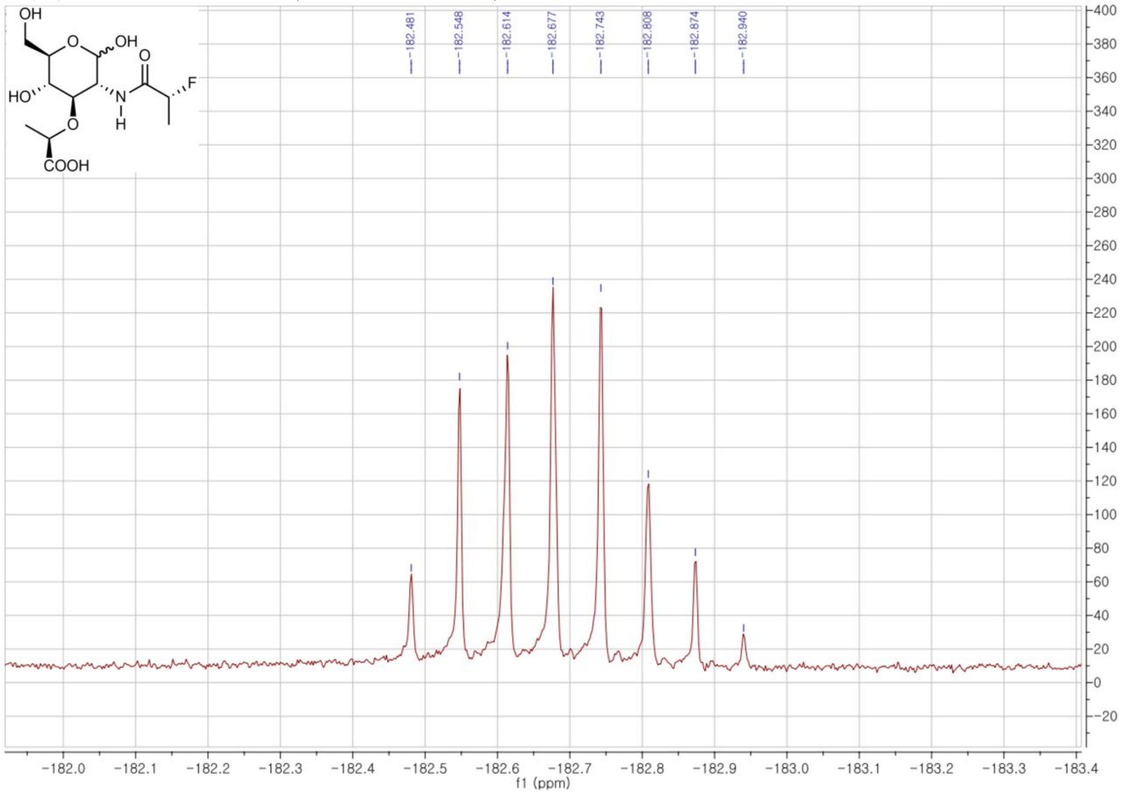

B.6.1. (S)-4-Nitrophenyl 2-(tosyloxy)propanoate ^{1}H NMR (400 MHz, $CDCl_3$)

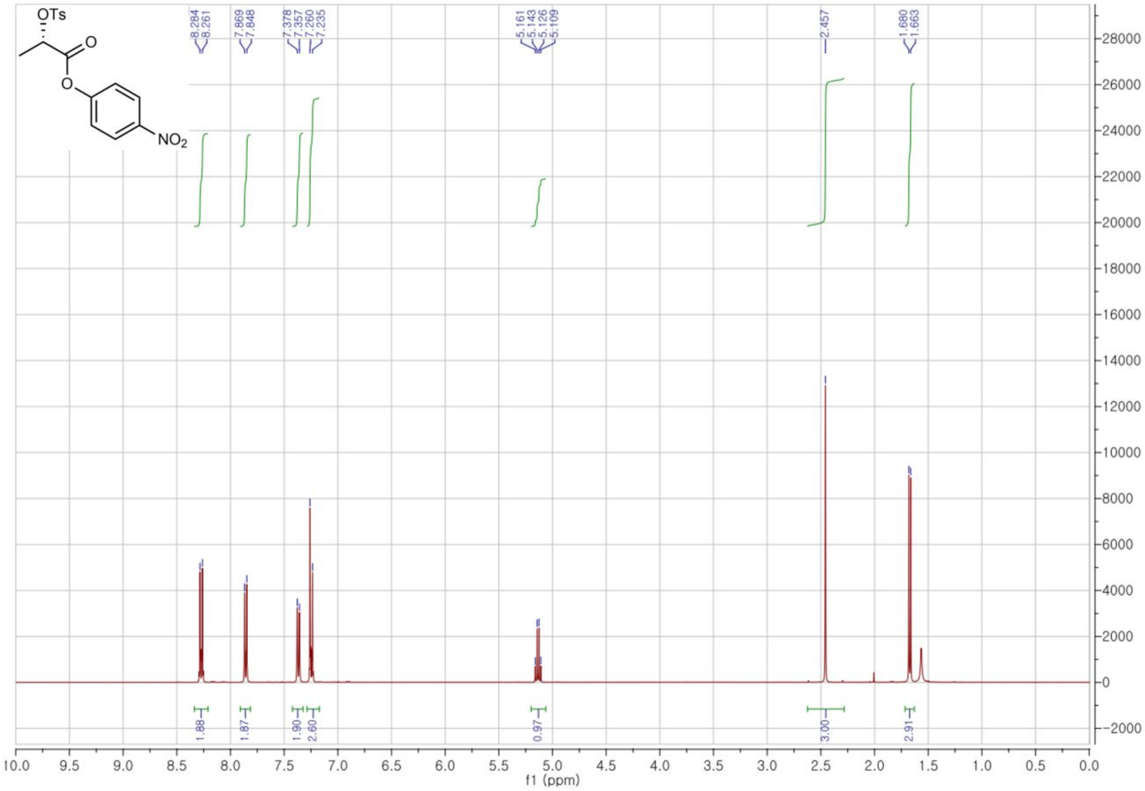

B.6.2. (S)-4-Nitrophenyl 2-(tosyloxy)propanoate ^{13}C NMR (100 MHz, $CDCl_3$)

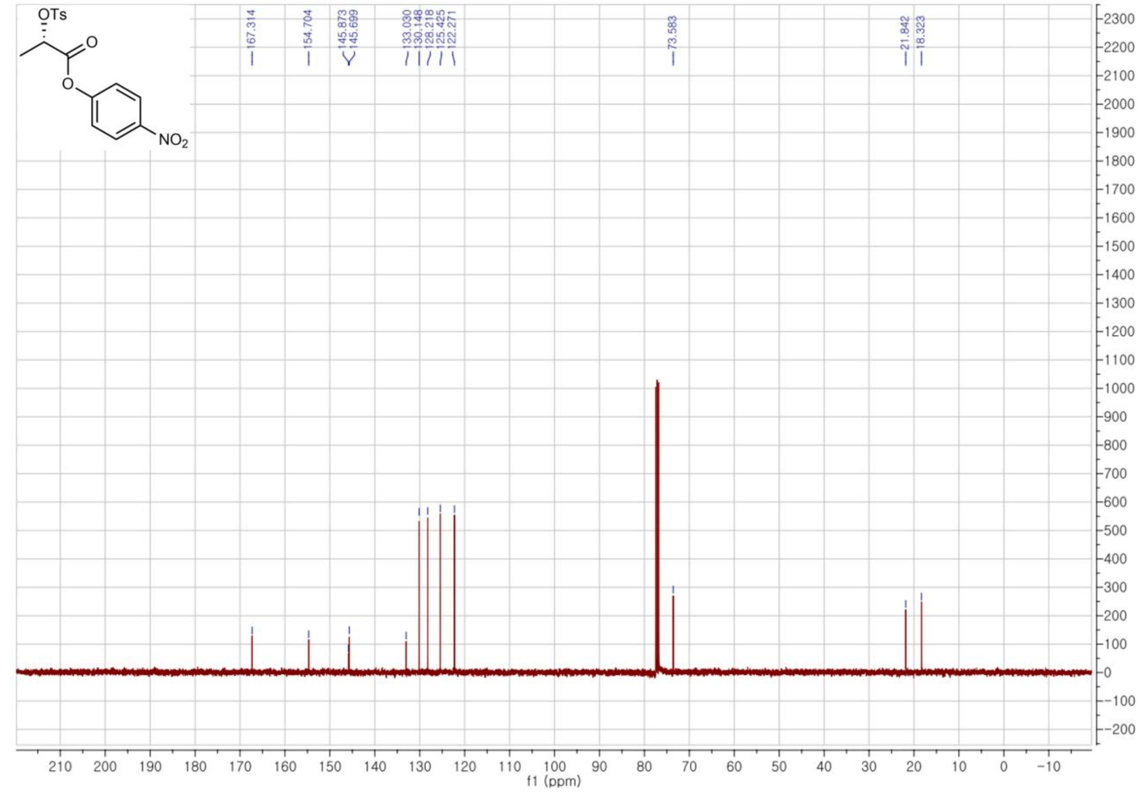

C. Supplemental Figures and Table

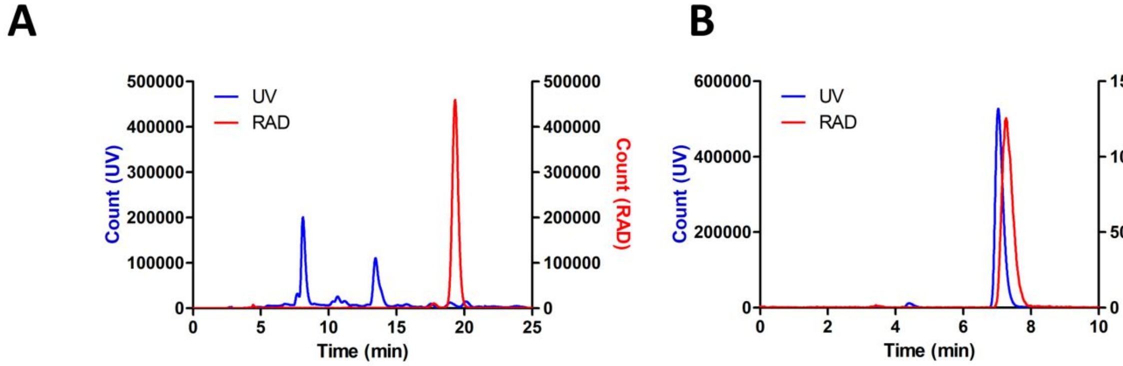

Fig. S1. HPLC profiles of [^{18}F]NFP. (A) Representative semi-preparative HPLC profile (Phenomenex; Luna 10 µm C18 250x10 mm; 45% ACN/Water containing 0.1% TFA; 4 mLmin^{-1}) for the purification of [^{18}F]NFP. (B) Analytical-HPLC profile (Phenomenex; Luna 10 µm C18 250x4.6 mm; 60% ACN/Water containing 0.1% TFA; 1 mLmin^{-1}) of [^{18}F]NFP co-injected with ^{19}F standard.

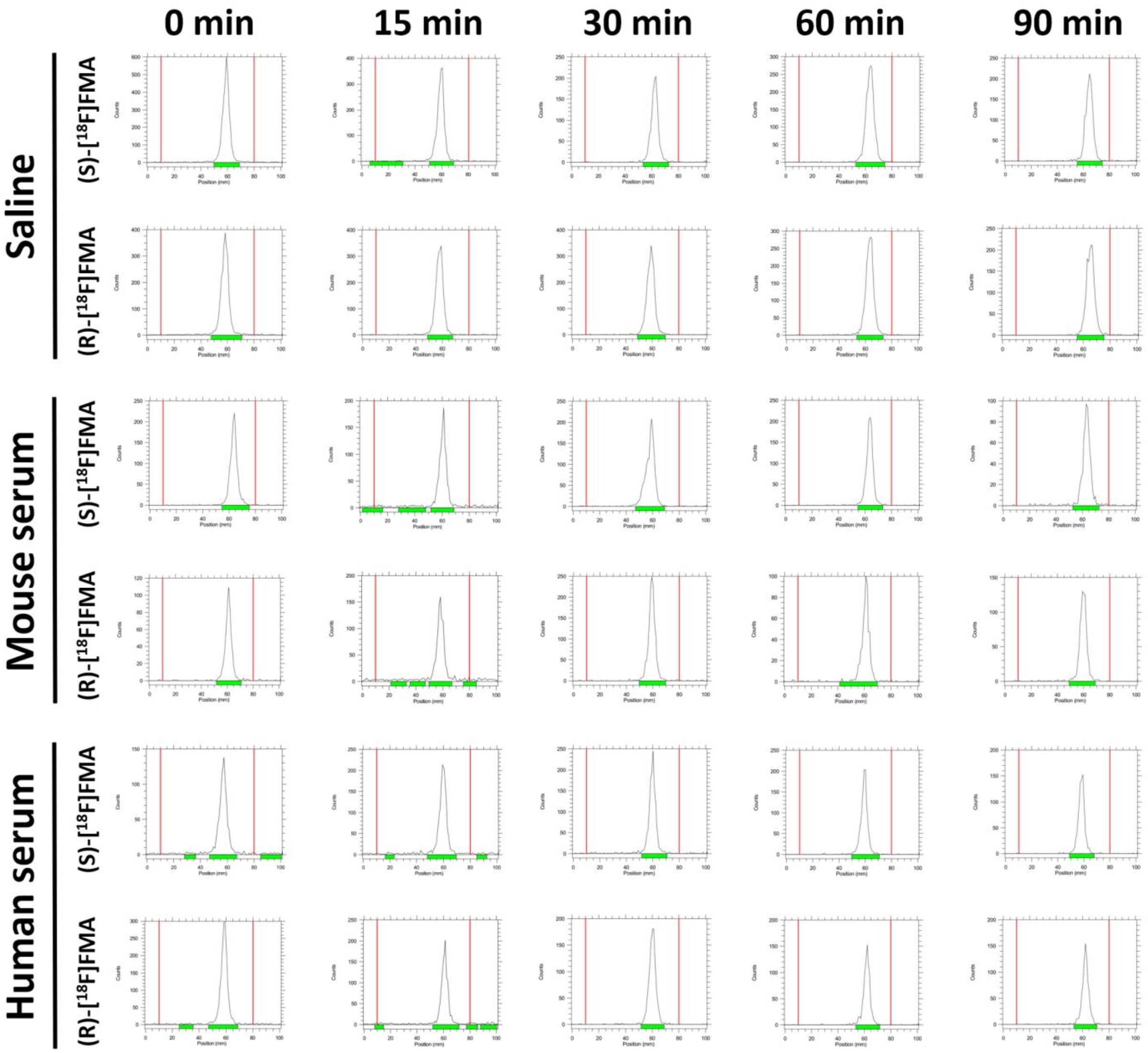

Fig. S2. Representative radio-TLC profiles of (S)- and (R)-[^{18}F]FMA in saline, mouse serum, human serum at 37 ^{o}C for 0, 15, 30, 60, and 90 min, respectively. There were no significant difference R_f values between 4 different diastereomers of [^{18}F]FMA .

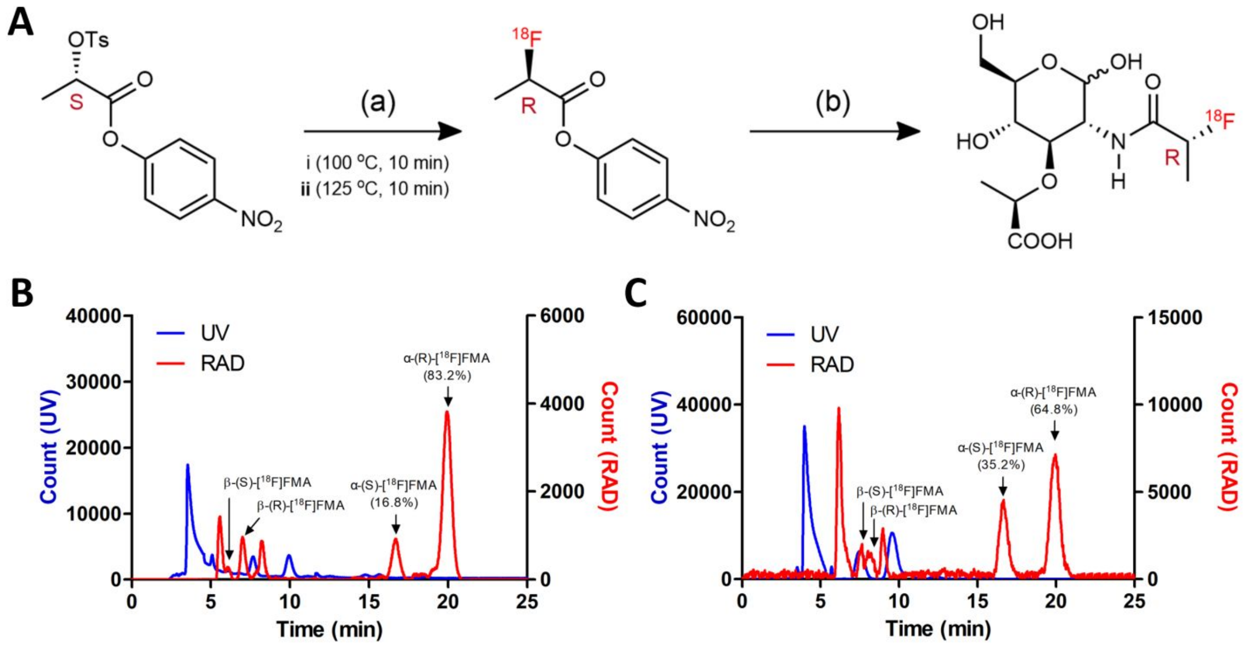

Fig. S3. (A) Scheme for the radiosynthesis of (R)-[^{18}F]FMA from (S)-NOTsP (**8**) precursor via 2 steps. Reagent and conditions: (a) precursor (**8**, 8 mg, 29.2 µmol), $^{18}F^-$, Cs_2CO_3 (2.2 mg, 6.8 µmol), 80% tBuOH/MeCN (0.5 mL), 10 min at i) 100 ^{o}C or ii) 125 ^{o}C. (b) α-muramic acid (0.25 mg, 1.0 µmol), 0.1% TEA/DMSO (100 µL), 60 ^{o}C. Semi-preparative HPLC profiles (Phenomenex; Luna 10 µm C18 250x10 mm; 5% EtOH/Water containing 0.1% HCl; 4 mLmin^{-1}) after coupling reaction of muramic acid with [^{18}F]NFP which was prepared from condition **i** (B) and **ii** (C).

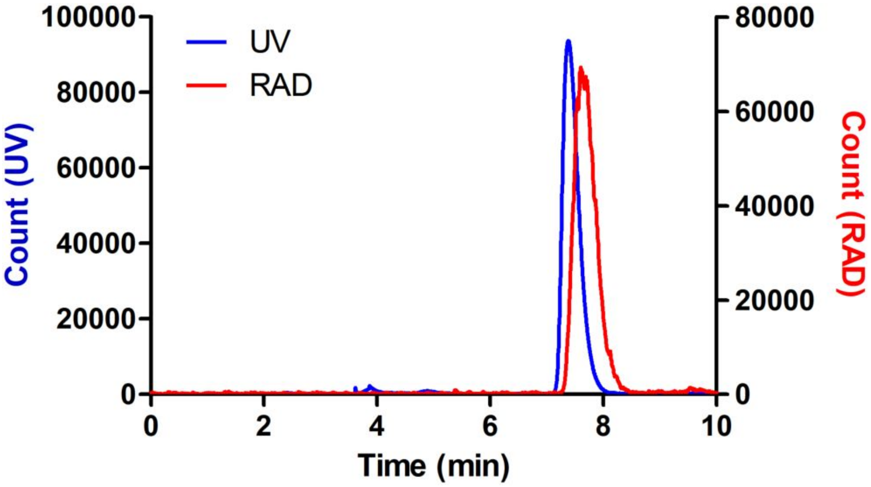

Fig. S4. Analytical-HPLC profile (Phenomenex; Luna 10 µm C18 250x4.6 mm; 10% EtOH/Water containing 0.1% HCl; 1 $mLmin^{-1}$) of [^{18}F]FPA co-injected with a cold standard.

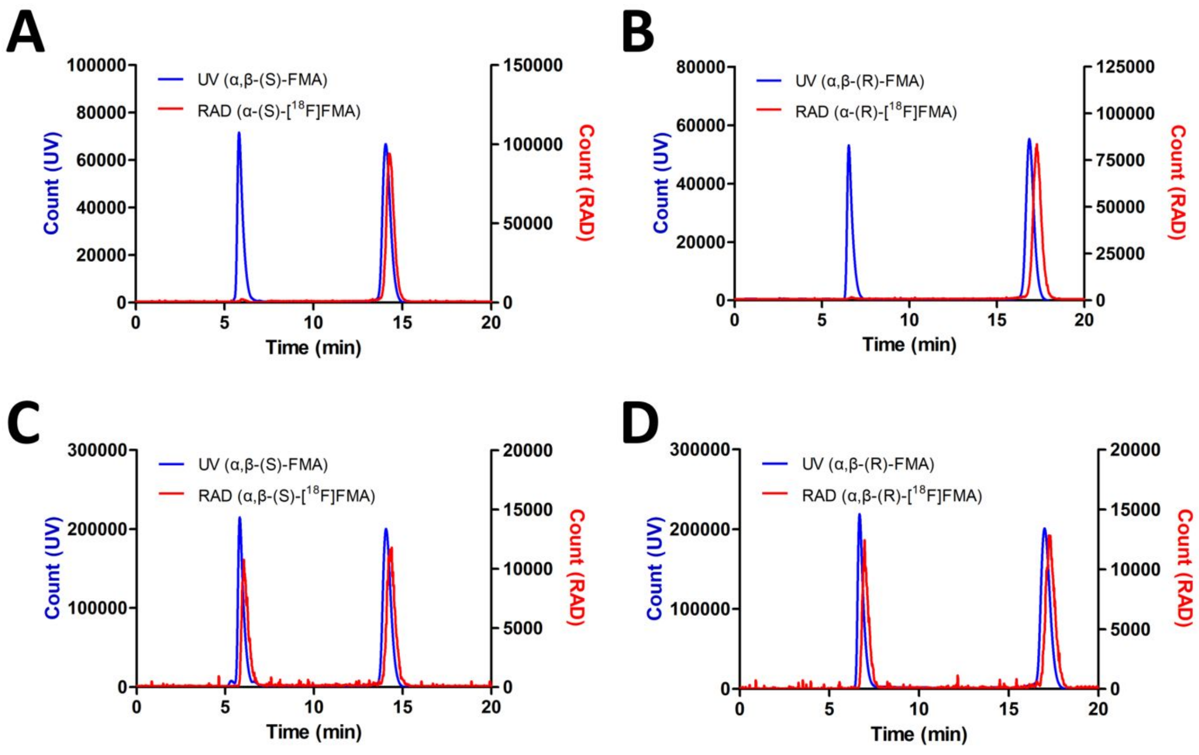

Fig. S5. Analytical-HPLC profiles (Phenomenex; Luna 10μm C18 250x4.6 mm; 5% EtOH/Water containing 0.1% HCl; 1 mLmin^{-1}) of (R,S)-[^{18}F]FMA co-injected with a cold standard at different time. (A) (S)-[^{18}F]FMA after HPLC purification. (B) (R)-[^{18}F]FMA after HPLC purification. (C) (S)-[^{18}F]FMA 4 h post HPLC purification. (D) (R)-[^{18}F]FMA 4 h post HPLC purification.

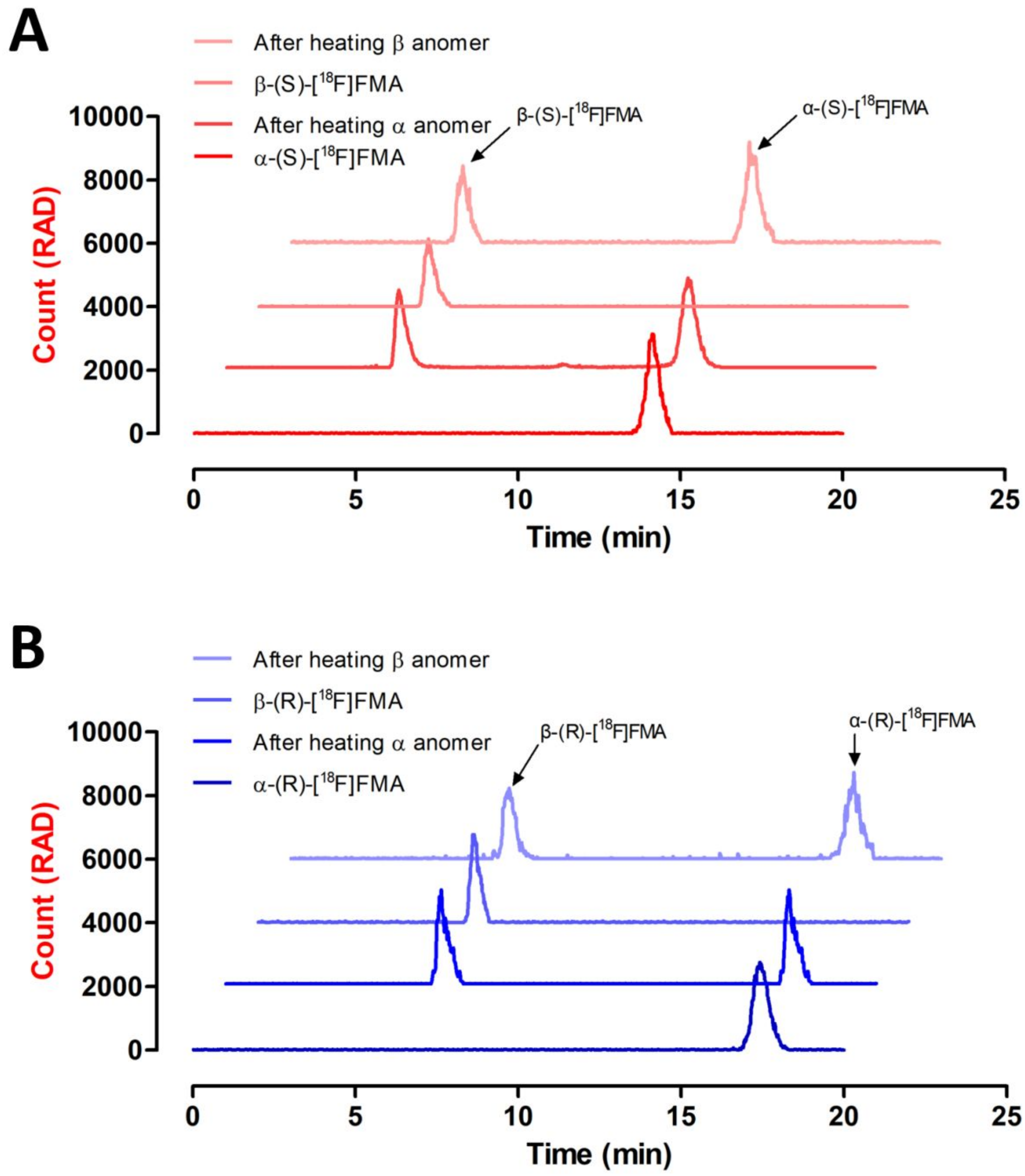

Fig. S6. Heat-promoted mutarotation analysis of (S)- and (R)-[^{18}F]FMA. Analytical HPLC profile (Phenomenex; Luna 10 µm C18 250x4.6 mm; 5% EtOH/Water containing 0.1% HCl; 1 mLmin^{-1}) of (S)-[^{18}F]FMA (A) and (R)-[^{18}F]FMA (B) before and after heating at 90 ^{o}C for 10 min.

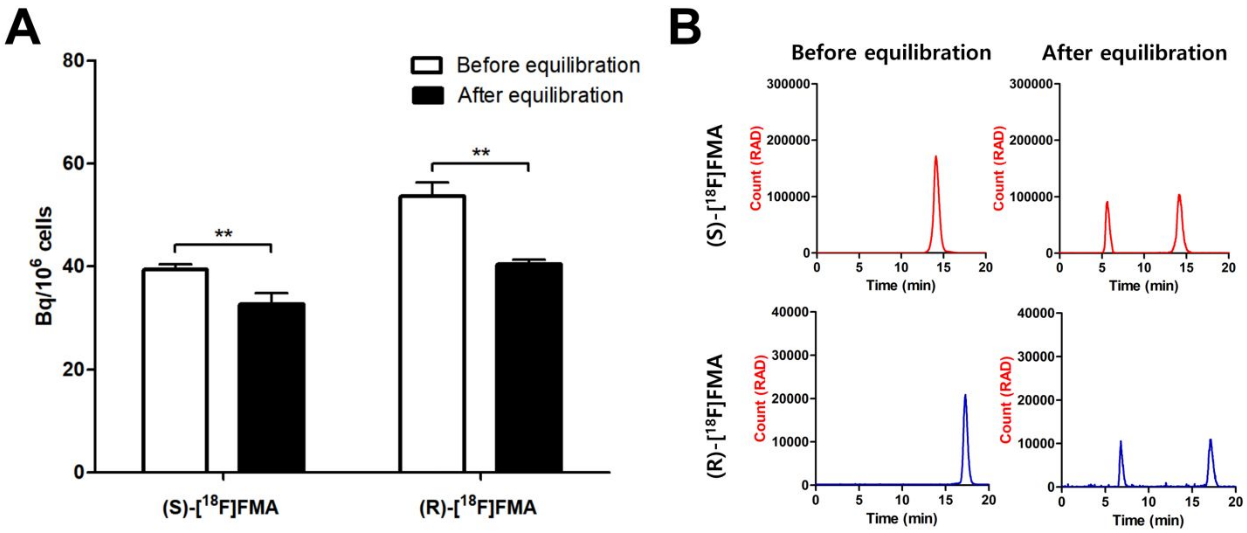

Fig. S7. *In vitro* analyses of (*S*)-[^{18}F]FMA and (*R*)-[^{18}F]FMA in S. aureus before (A) and after α-β equilibration (B). $^{**}P<0.01$

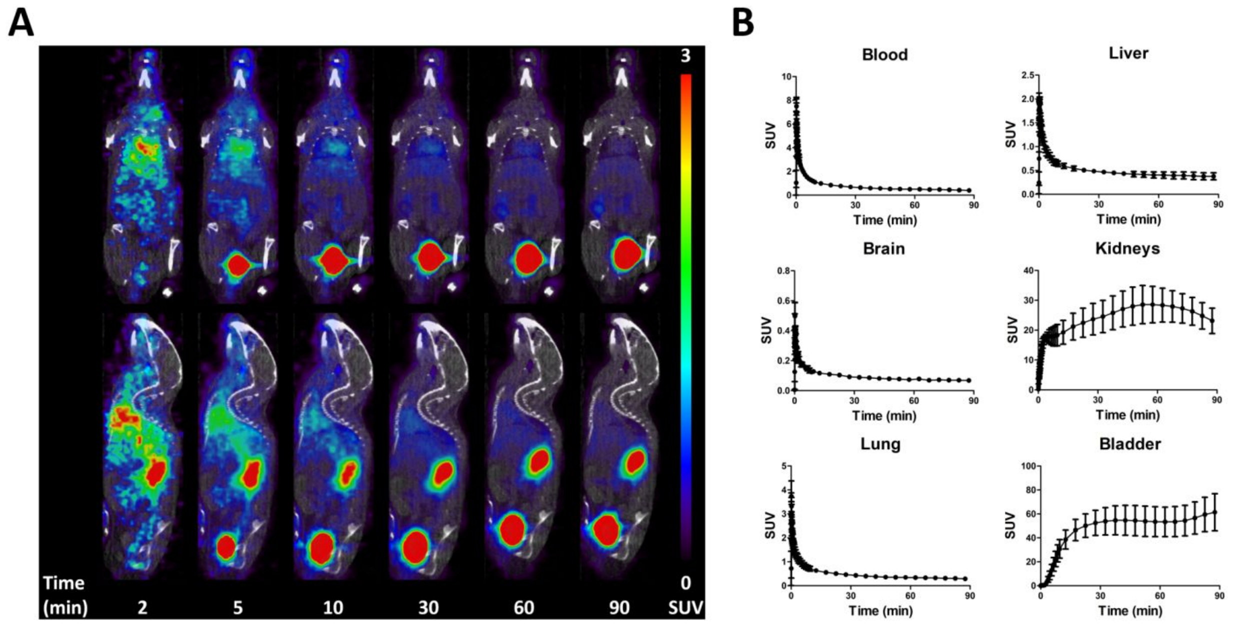

C

	Blood	Brain	Lung	Liver	Kidneys	Bladder
$T_{1/2\alpha}$	1.3±0.5	1.5±0.5	1.4±0.5	1.3±0.6	n.d.	n.d.
$T_{1/2\beta}$	15.0±9.7	19.1±8.8	13.0±5.0	17.2±10.4	n.d.	n.d.
C_{max}	8.5 ± 2.1	0.6 ± 0.2	4.6 ± 1.8	2.0 ± 0.4	30.0 ± 12.7	n.d.
T_{max}	0.2 ± 0.1	0.2 ± 0.1	0.2 ± 0.0	0.3 ± 0.1	65.0 ± 12	n.d.
AUC	66.47 ± 16.12	8.298 ± 1.382	42.26 ± 11.93	43.57 ± 10.50	2136 ± 812.4	4224 ± 1815

Fig. S8. Dynamic μPET/CT imaging analysis of (*R*)-[^{18}F]FMA in healthy mice (n = 4). (A) Representative time-course PET/CT imaging of (*R*)-[^{18}F]FMA in a healthy mouse. (B) Time-activity curves of (*R*)-[^{18}F]FMA in organs of healthy mice. (C) Kinetic parameters of (R)-[^{18}F]FMA in normal mice. SUV: Standardized uptake value, $T_{1/2}$: half-life (min, distribution (α) and elimination (β), respectively), C_{max}: peak concentration (SUV), T_{max}: time at C_{max} (min), AUC: area under the curve (SUV·min), n.d.: not determined.

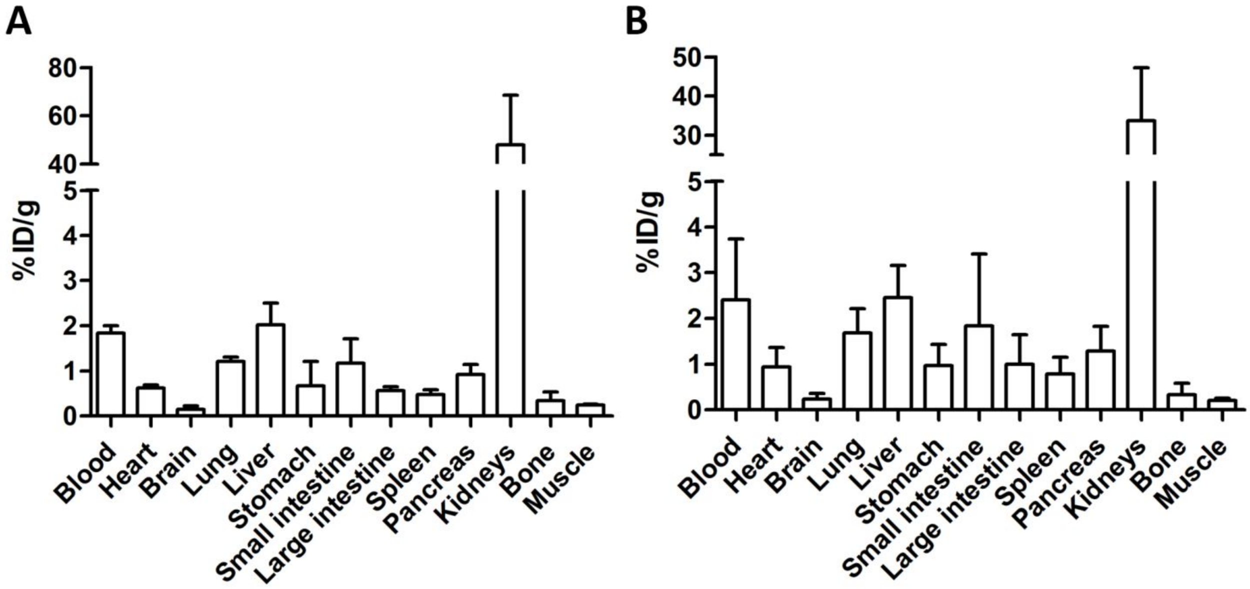

Fig. S9. *Ex vivo* biodistribution of (*S*)-[^{18}F]FMA (A) and (*R*)-[^{18}F]FMA (B) in healthy mice (n = 4 for each). *Ex vivo* biodistribution analysis was conducted immediately after μPET/CT imaging studies by harvesting the organs and tissues using a Hidex Automatic Gamma Counter.

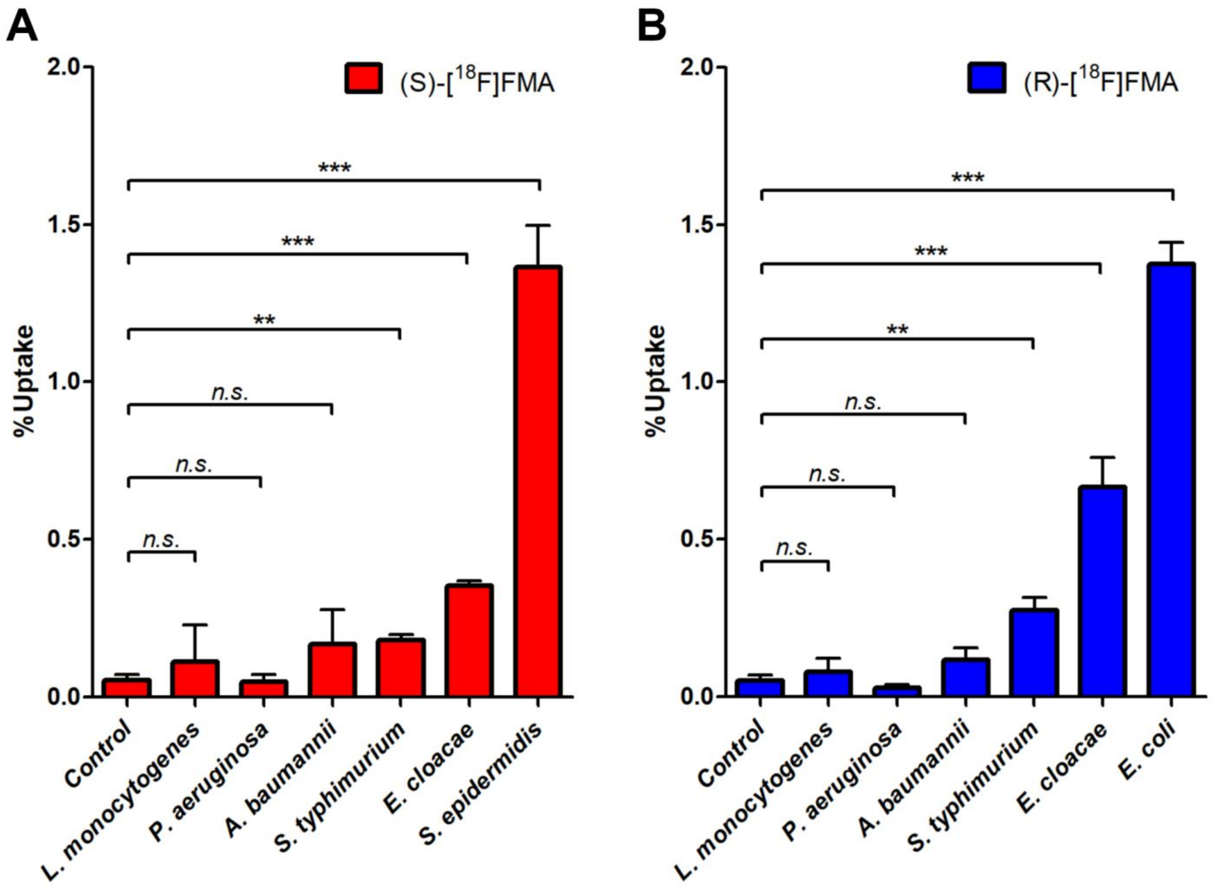

Fig. S10. *In vitro* cellular uptake of (*S*)-[^{18}F]FMA (A) and (*R*)-[^{18}F]FMA (B) compared with the control (non-specific binding of the tracers in the filter membrane). ***P*<0.01, ****P*<0.001, *n.s.*: not significant.

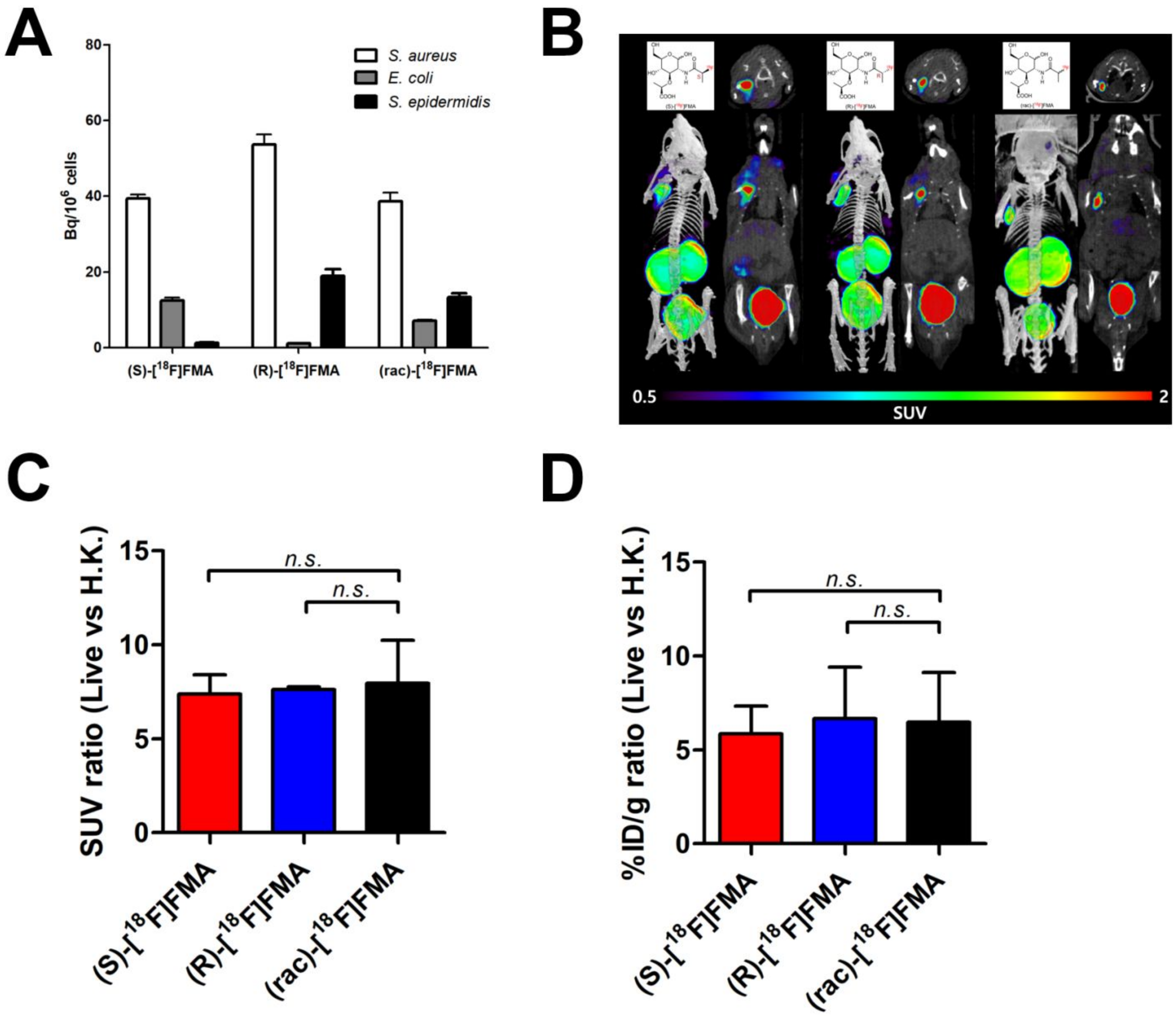

Fig. S11. Comparison of *in vitro and in vivo* detection sensitivity of the tracers in *S. aureus*. (A) *In vitro* cellular uptake of (*S*)-[^{18}F]FMA, (*R*)-[^{18}F]FMA, and (*rac*)-[^{18}F]FMA in *S. aureus*. (B) Representative µPET/CT images (obtained from the last frame of imaging data; 85 – 90 min) of (*S*)-[^{18}F]FMA, (*R*)-[^{18}F]FMA, and (*rac*)-[^{18}F]FMA in mice. (C) µPET ROI-derived SUV ratio (live vs. H.K.) in infected mice (obtained from the last frame of imaging data; 85 – 90 min). (D) *Ex vivo* %IDg^{-1} ratio (live vs. H.K.) in infected mice.

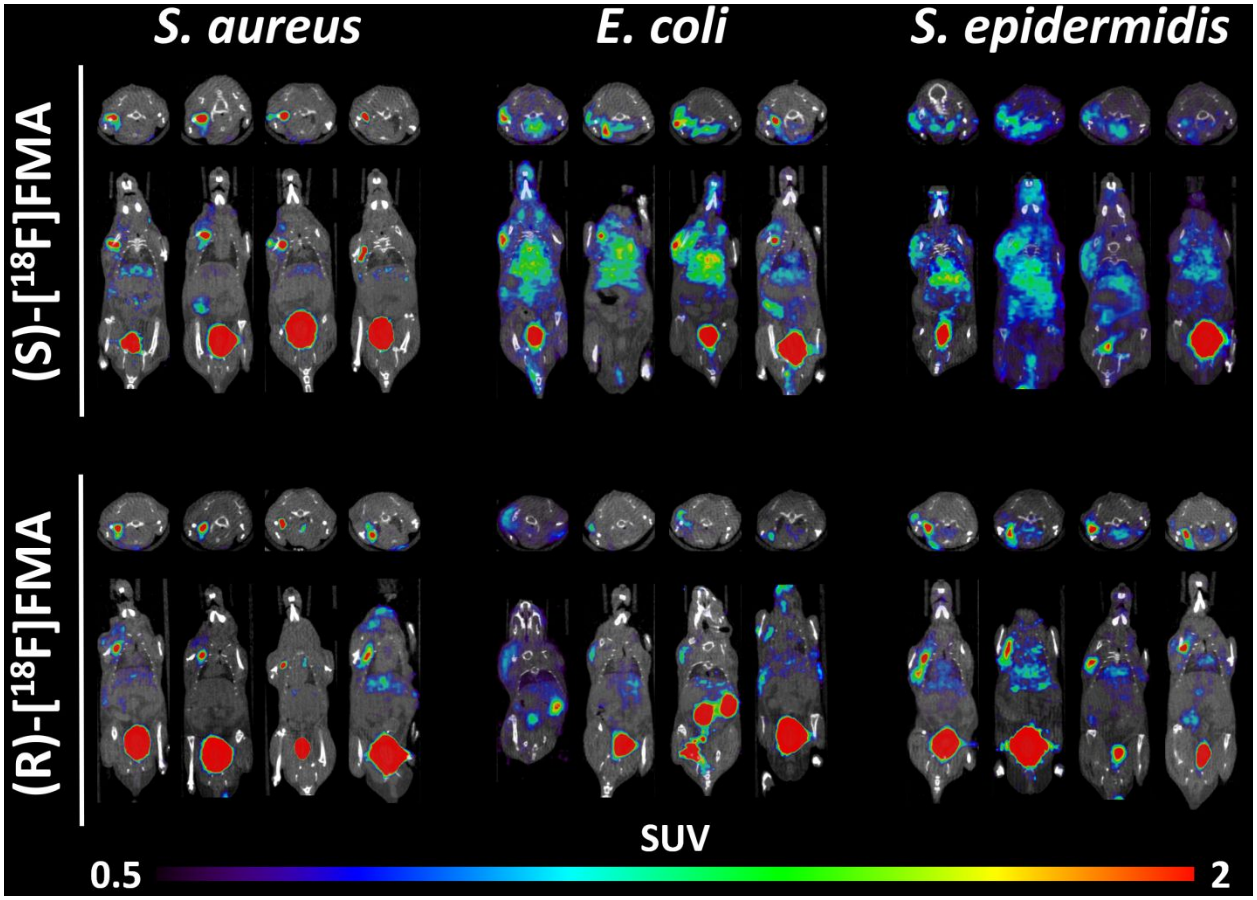

Fig. S12. Dynamic μPET/CT imaging analysis of (*S*)- and (*R*)-[^{18}F]FMA in murine myositis models 90 min post-injection.

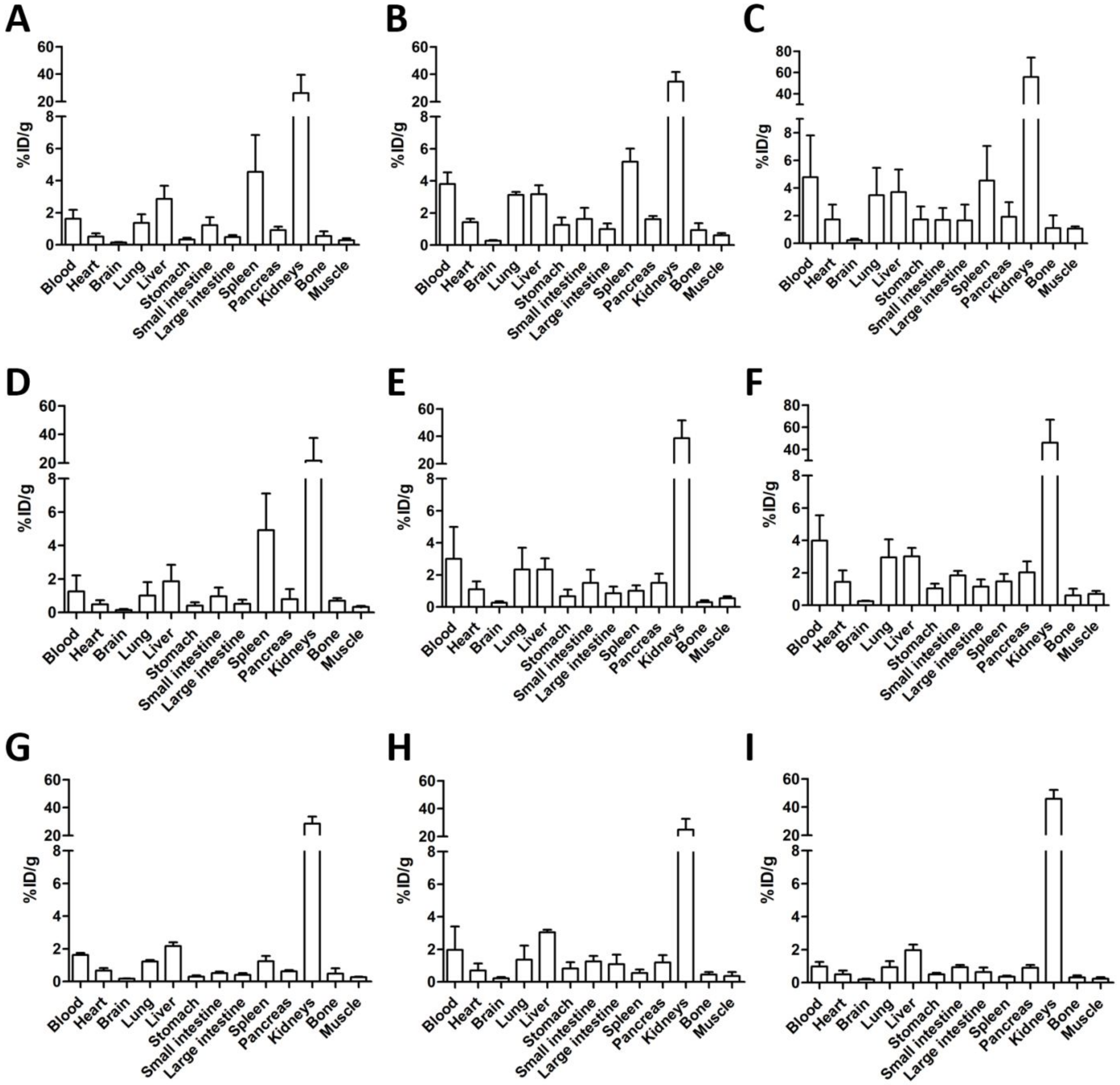

Fig. S13. *Ex vivo* biodistribution of (*S*)- and (*R*)-[^{18}F]FMA in murine myositis models (n = 4 for each). *Ex vivo* biodistribution data of (*S*)-[^{18}F]FMA in *S. aureus* (*A*), *E. coli* (*B*), and *S. epidermidis* (C) inoculating models respectively. *Ex vivo* biodistribution data of (*R*)-[^{18}F]FMA in *S. aureus* (D), *E. coli* (E), and *S. epidermidis* (F) inoculating models respectively. *Ex vivo* biodistribution data of (*R*)-[^{18}F]FMA in *S. aureus* (G; 10^4 inoculated, H; 10^5 inoculated) and (*rac*)-[^{18}F]FMA in *S. aureus* (I) inoculating models respectively. *Ex vivo* biodistribution analysis was conducted immediately after μPET/CT imaging studies by harvesting the organs and tissues using a Hidex Automatic Gamma Counter.

Table S1. Bacteria strains and growth conditions used for this study.

Bacteria Strain	Phenotype or Genotype	Source or Reference	Growth Media
S. aureus	Wild-type	ATCC 12600	Luria Broth
S. aureus (Xen29)	ATCC 12600 expressing the *Photorhabdus luminescens luxABCDE* genes	Xenogen USA	Luria Broth
Methicillin-resistant S. aureus	MRSA Clinical isolate	University of Nebraska Medical Center	Luria Broth
S. epidermidis	Wild-type	ATCC 35984	Luria Broth
E. faecalis	Wild-type	ATCC 19433	Brain Heart Infusion Broth
L. monocytogenes	Wild-type	ATCC 15313	Brain Heart Infusion Broth
E. coli	Wild-type	ATCC 25922	Luria Broth
E. coli (Xen14)	E. coli WS2572 with an stable copy of the Photorhabdus luminescens lux operon on the bacterial chromosome	Xenogen USA	Luria Broth
P. aeruginosa	Wild-type	ATCC 10145	Luria Broth
P. aeruginosa (Xen41)	PA01 with an stable copy of the Photorhabdus luminescens lux operon on the bacterial chromosome	Xenogen USA	Luria Broth
K. pneumoniae	Wild-type	ATCC 13883	Luria Broth
P. mirabilis	Wild-type	ATCC 29906	Luria Broth
A. baumannii	Wild-type	ATCC 19606	Luria Broth
S. typhimurium	Wild-type	ATCC 29630	Luria Broth
E. cloacae	Wild-type	ATCC 7256	Luria Broth

D. Reference

1. A. V. Kurkin, D. S. Belov, and M. A. Yurovskaya. tert-Butyl (2S)-(p-tolylsulfonyloxy)-propionate – a suitable reagent for the direct alkylation of indole derivatives. Chem. Heterocycl. 2008,44(9);1123-1128.
